# Large scale variation in the rate of germ-line *de novo* mutation, base composition, divergence and diversity in humans

**DOI:** 10.1371/journal.pgen.1007254

**Published:** 2018-03-28

**Authors:** Thomas C. A. Smith, Peter F. Arndt, Adam Eyre-Walker

**Affiliations:** 1 School of Life Sciences, University of Sussex, Brighton, United Kingdom; 2 Max Planck Institute for Molecular Genetics, Berlin, Germany; Brigham and Women's Hospital, Harvard Medical School, UNITED STATES

## Abstract

It has long been suspected that the rate of mutation varies across the human genome at a large scale based on the divergence between humans and other species. However, it is now possible to directly investigate this question using the large number of *de novo* mutations (DNMs) that have been discovered in humans through the sequencing of trios. We investigate a number of questions pertaining to the distribution of mutations using more than 130,000 DNMs from three large datasets. We demonstrate that the amount and pattern of variation differs between datasets at the 1MB and 100KB scales probably as a consequence of differences in sequencing technology and processing. In particular, datasets show different patterns of correlation to genomic variables such as replication time. Never-the-less there are many commonalities between datasets, which likely represent true patterns. We show that there is variation in the mutation rate at the 100KB, 1MB and 10MB scale that cannot be explained by variation at smaller scales, however the level of this variation is modest at large scales–at the 1MB scale we infer that ~90% of regions have a mutation rate within 50% of the mean. Different types of mutation show similar levels of variation and appear to vary in concert which suggests the pattern of mutation is relatively constant across the genome. We demonstrate that variation in the mutation rate does not generate large-scale variation in GC-content, and hence that mutation bias does not maintain the isochore structure of the human genome. We find that genomic features explain less than 40% of the explainable variance in the rate of DNM. As expected the rate of divergence between species is correlated to the rate of DNM. However, the correlations are weaker than expected if all the variation in divergence was due to variation in the mutation rate. We provide evidence that this is due the effect of biased gene conversion on the probability that a mutation will become fixed. In contrast to divergence, we find that most of the variation in diversity can be explained by variation in the mutation rate. Finally, we show that the correlation between divergence and DNM density declines as increasingly divergent species are considered.

## Author summary

Using a dataset of more than 130,000 *de novo* mutations we show that there is large-scale variation in the mutation rate at the 100KB and 1MB scales. We show that different types of mutation vary in concert and in a manner that is not expected to generate variation in base composition; hence mutation bias is not responsible for the large-scale variation in base composition that is observed across human chromosomes. As expected, large-scale variation in the rate of divergence between species and the variation within species across the genome, are correlated to the rate of mutation, but the correlation between divergence and the mutation rate is not as strong as it could be. We show that biased gene conversion is responsible for weakening the correlation. In contrast, we find that most of the variation across the genome in diversity can be explained by variation in the mutation rate. Finally, we show that the correlation between the rate of mutation in humans and the divergence between humans and other species, weakens as the species become more divergent.

## Introduction

Until recently, the distribution of germ-line mutations across the genome was studied using patterns of nucleotide substitution between species in putatively neutral sequences (see [1] for review of this literature), since under neutrality the rate of substitution should be equal to the mutation rate. However, the sequencing of hundreds of individuals and their parents has led to the discovery of thousands of germ-line *de novo* mutations (DNMs) in humans [2–6]; it is therefore possible to analyse the pattern of DNMs directly rather than inferring their patterns from substitutions. Initial analyses have shown that the rate of germ-line DNM increases with paternal age [4], a result that was never-the-less inferred by Haldane some 70 years ago [7], maternal age [6], varies across the genome [5] and is correlated to a number of factors, including the time of replication [3], the rate of recombination [3], GC content [5] and DNA hypersensitivity [5].

Previous analyses have demonstrated that there is large scale (e.g. 1MB) variation in the rate of DNM in both the germ-line [3, 5] and the somatic tissue [8–12]. Here we focus exclusively on germ-line mutations. We use a collection of over 130,000 germ-line DNMs to address a range of questions pertaining to the large-scale distribution of DNMs. First, we quantify how much variation there is at different scales and investigate whether the variation in the mutation rate at a large-scale can be explained in terms of variation at smaller scales. We also investigate to what extent the variation is correlated between different types of mutation, and to what extent it is correlated to a range of genomic variables.

We use the data to investigate a long-standing question–what forces are responsible for the large-scale variation in GC content across the human genome, the so called “isochore” structure [13]. It has been suggested that the variation could be due to mutation bias [14–18], natural selection [13, 19, 20], biased gene conversion [21–24], or a combination of all three forces [25]. There is now convincing evidence that biased gene conversion plays a role in the generating at least some of the variation in GC-content [26–28]. However, this does not preclude a role for mutation bias or selection. With a dataset of DNMs we are able to directly test whether mutation bias causes variation in GC-content.

The rate of divergence between species is known to vary across the genome at a large scale [1]. As expected this appears to be in part due to variation in the rate of mutation [3]. However, the rate of mutation at the MB scale is not as strongly correlated to the rate of nucleotide substitution between species as it could be if all the variation in divergence between 1MB windows was due to variation in the mutation rate [3]. Instead, the rate of divergence appears to correlate independently to the rate of recombination. This might be due to one, or a combination, of several factors. First, recombination might affect the probability that a mutation becomes fixed by the process of biased gene conversion (BGC) (reviewed by [26]). Second, recombination can affect the probability that a mutation will be fixed by natural selection; in regions of high recombination deleterious mutations are less likely to be fixed, whereas advantageous mutations are more likely. Third, low levels of recombination can increase the effects of genetic hitch-hiking and background selection, both of which can reduce the diversity in the human-chimp ancestor, and the time to coalescence and the divergence between species. There is evidence of this effect in the divergence of humans and chimpanzees, because the divergence between these two species is lower nearer exons and other functional elements [29, 30]. And fourth, the correlation of divergence to both recombination and DNM density might simply be due to limitations in multiple regression; spurious associations can arise if multiple regression is performed on two correlated variables that are subject to sampling error. For example, it might be that divergence only depends on the mutation rate, but that the mutation rate is partially dependent on the rate of recombination. In a multiple regression, divergence might come out as being correlated to both DNM density and the recombination rate, because we do not know the mutation rate without error, since we only have limited number of DNMs. Here, we introduce a test that can resolve between these explanations.

As with divergence, we might expect variation in the level of diversity across a genome to correlate to the mutation rate. The role of the mutation rate variation in determining the level of genetic diversity across the genome has long been a subject of debate. It was noted many years ago that diversity varies across the human genome at a large scale and that this variation is correlated to the rate of recombination [31–33]. Because the rate of substitution between species is also correlated to the rate of recombination, Hellmann et al. [31, 32] inferred that the correlation between diversity and recombination was at least in part due to a mutagenic effect of recombination, an inference that has been confirmed by recent studies of recombination [3, 34, 35]. However, no investigation has been made as to whether variation in the rate of mutation explains all the variation in diversity, or whether biased gene conversion, direct and linked selection have a major influence on diversity at a large scale.

## Results

### De novo mutations

To investigate large scale patterns of *de novo* mutation in humans we compiled data from three studies which between them had discovered more than 130,000 autosomal DNMs: 105,385 from Jonsson et al. [36], 26,939 mutations from Wong et al. [6], and 11016 mutations from Francioli et al. [3] The datasets are henceforth referred to by the name of the first author. We divided the mutations up into 9 categories reflecting the fact that CpG dinucleotides have higher mutation rates than non-CpG sites, and the fact that we cannot differentiate which strand the mutation had occurred on: CpG C>T (a C to T or G to A mutation at a CpG site), CpG C>A, CpG C>G and for non-CpG sites C>T, T>C, C>A, T>G, C<>G and T<>A mutations.

**Fig 1:**
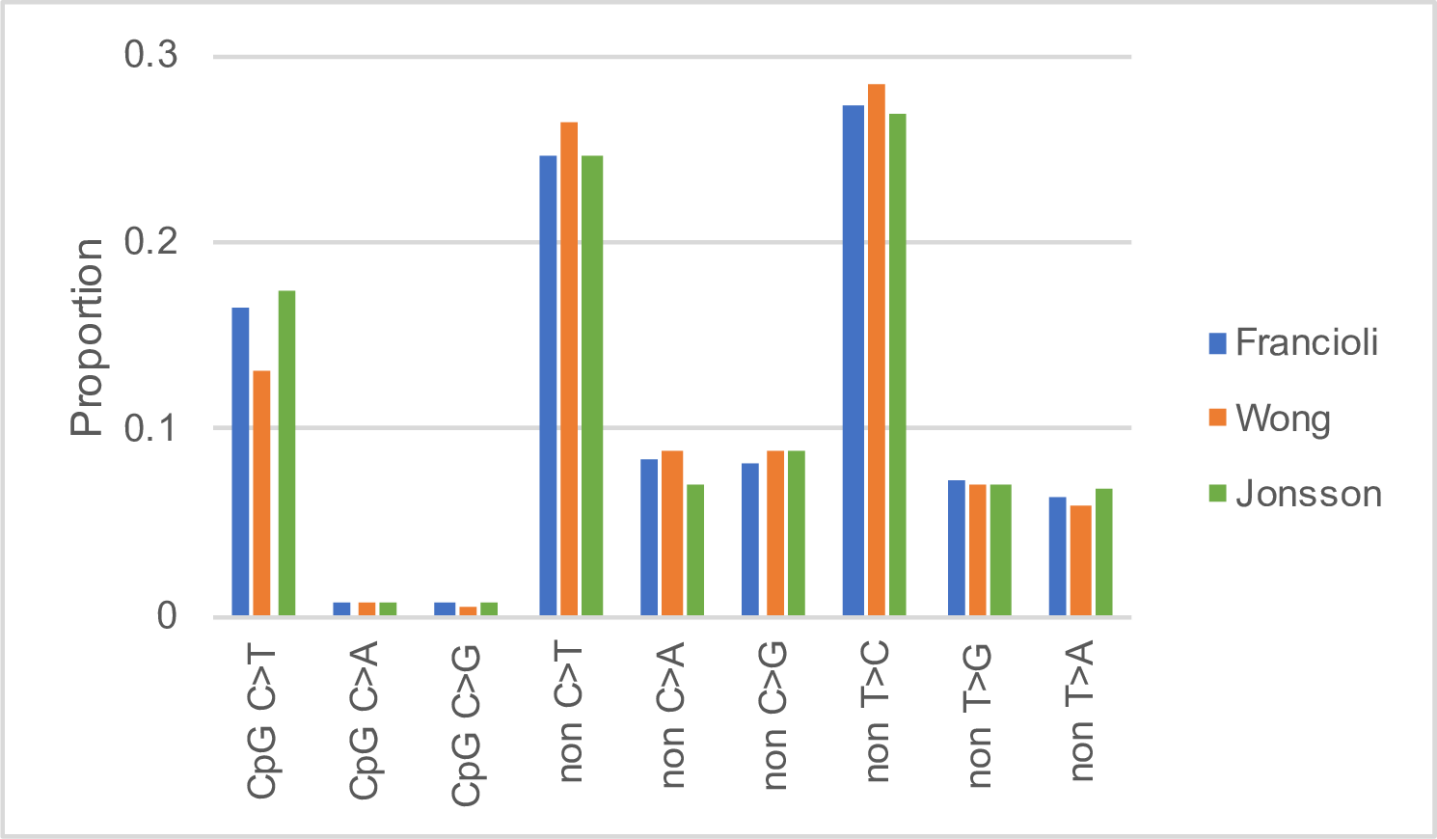
The proportion of DNMs in each mutational category in the three datasets. CpG X>Y is an X>Y DNM at a CpG site, non X>Y is an X>Y DNM at a non-CpG site.

The proportion of mutations in each category in each of the datasets is shown in Fig 1. We find that the pattern of mutation differs significantly between the studies (Chi-square test of independence on the number of mutations in each of the 9 categories, p < 0.0001). This appears to be largely due to the relative frequency of C>T transitions in both the CpG and non-CpG context; a discrepancy which has been noted before[37, 38]. In the data from Wong et al. [6] the frequency of C>T transitions at CpG sites is ~13% whereas it is ~16–17% in the other two datasets. For non-CpG sites the frequency of C>T transitions is ~24% in all studies except that of Wong et al. in which it is 26%. It is not clear whether these patterns reflect differences in the mutation rate between different cohorts of individuals, possibly because of age [3, 4, 6] or geographical origin [39] or whether the differences are due to methodological problems associated with detecting DNMs.

### Distribution of rates

To investigate whether there is large scale variation in the mutation rate we divided the genome into non-overlapping windows of 10KB, 100KB, 1MB and 10MB and fit a gamma distribution to the number of mutations per region, taking into account the sampling error associated with the low number of mutations per region. We focussed our analysis at the 1MB scale since this has been extensively studied before. However, we show that the variation at 1MB forms part of a continuum of variation. We also repeated almost all our analyses at the 100KB scale with qualitatively similar results (these results are reported in supplementary tables).

**Fig 2:**
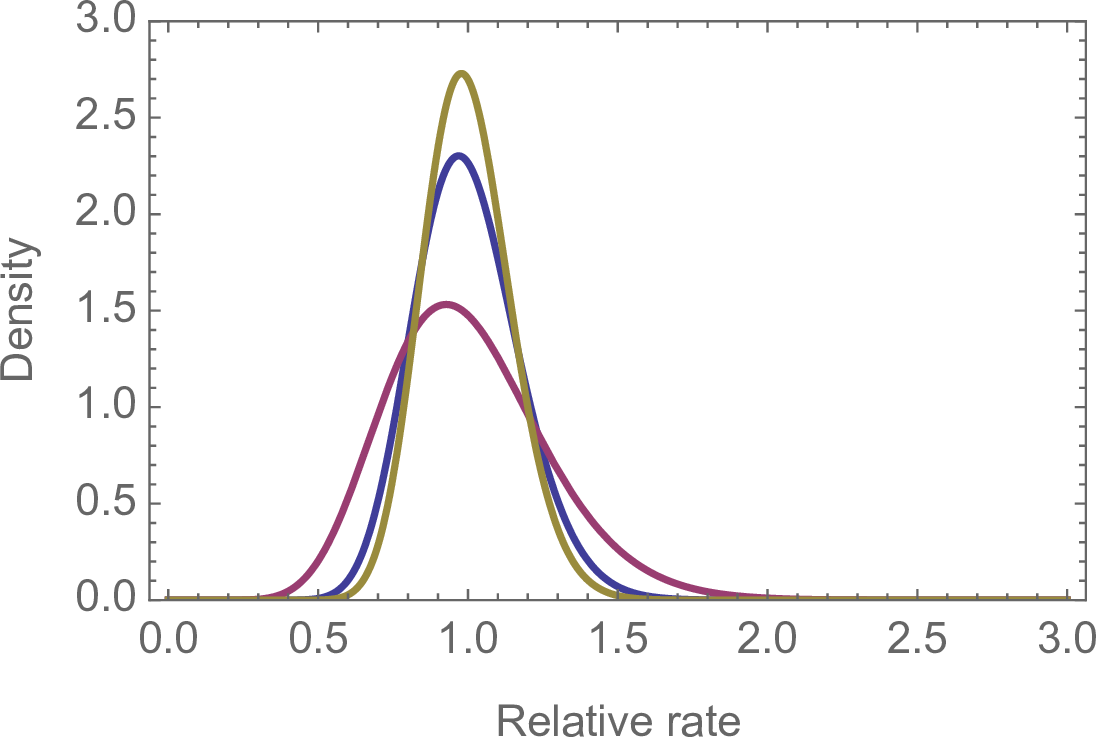
Gamma distributions fitted to the DNM density at the 1MB scale. In order of decreasing variance: Maroon–Wong, Blue–Francioli, Olive–Jonsson.

We find that the amount of variation differs significantly between the three studies (likelihood ratio tests: p < 0.001), although, the differences are quantitatively small at the 1MB (Fig 2) and 100KB (S1 Fig) scales. The variation between datasets might be due to differences in age or ethnicity between the individuals in each study, or methodological problems–for example, there might be differences between studies in the ability to identify DNMs. We can test whether callability is an issue in the Wong dataset because Wong et al. [6] estimated the number of trios at which a DNM was callable at each site. If we reanalyse the Wong data using the sum of the callable trios per MB, rather than the number of sites in the human genome assembly, we obtain very similar estimates of the distribution: the coefficient of variation (CV) for the distribution is 0.27 when we use the number of sites and 0.24 when we use the sum of callable trios.

As expected the number of DNMs per site is significantly correlated between the datasets (1MB Francioli v Wong r = 0.15, p<0.001; Francioli v Jonsson r = 0.19 p<0.001; Wong v Jonsson r = 0.29, p<0.001). The correlation is weak, but this is likely to be in part due to sampling error. If we simulate data assuming a common distribution, estimating the shape parameter as the mean CV of the distributions fit to the individual datasets, the mean simulated correlations are: Francioli v Wong r = 0.20; Francioli v Jonsson r = 0.29; Wong v Jonsson r = 0.41. This suggests that a substantial proportion of the variation is common to the three datasets, however in each case less than 5% of the simulated correlations are less than the observed correlation suggesting that some portion of the variation in the three datasets is uncorrelated.

The CV of the gamma distribution fitted to the density of DNMs is 0.18, 0.27 and 0.15 for the Francioli, Wong and Jonsson datasets respectively (Fig 2). The level of variation is significant (i.e. the lower 95% confidence interval of the CV is greater than zero), however the level of variation is modest (Fig 2). A gamma distribution with a coefficient of variation of 0.18 is one in which 90% of regions have a mutation rate within 30% of the mean (i.e. if the mean is one, between 0.7 and 1.3). The gamma distribution fits the distribution of rates qualitatively quite well (S2 Fig; S3 Fig for 100KB), even though a goodness-of-fit test rejects the model at both the 100KB and 1MB scales in all three datasets (p<0.001 in all cases). At the 1MB the observed distribution is more peaked than the fitted gamma distributed; there are too many regions with very low, very high and intermediate numbers of DNMs.

**Fig 3:**
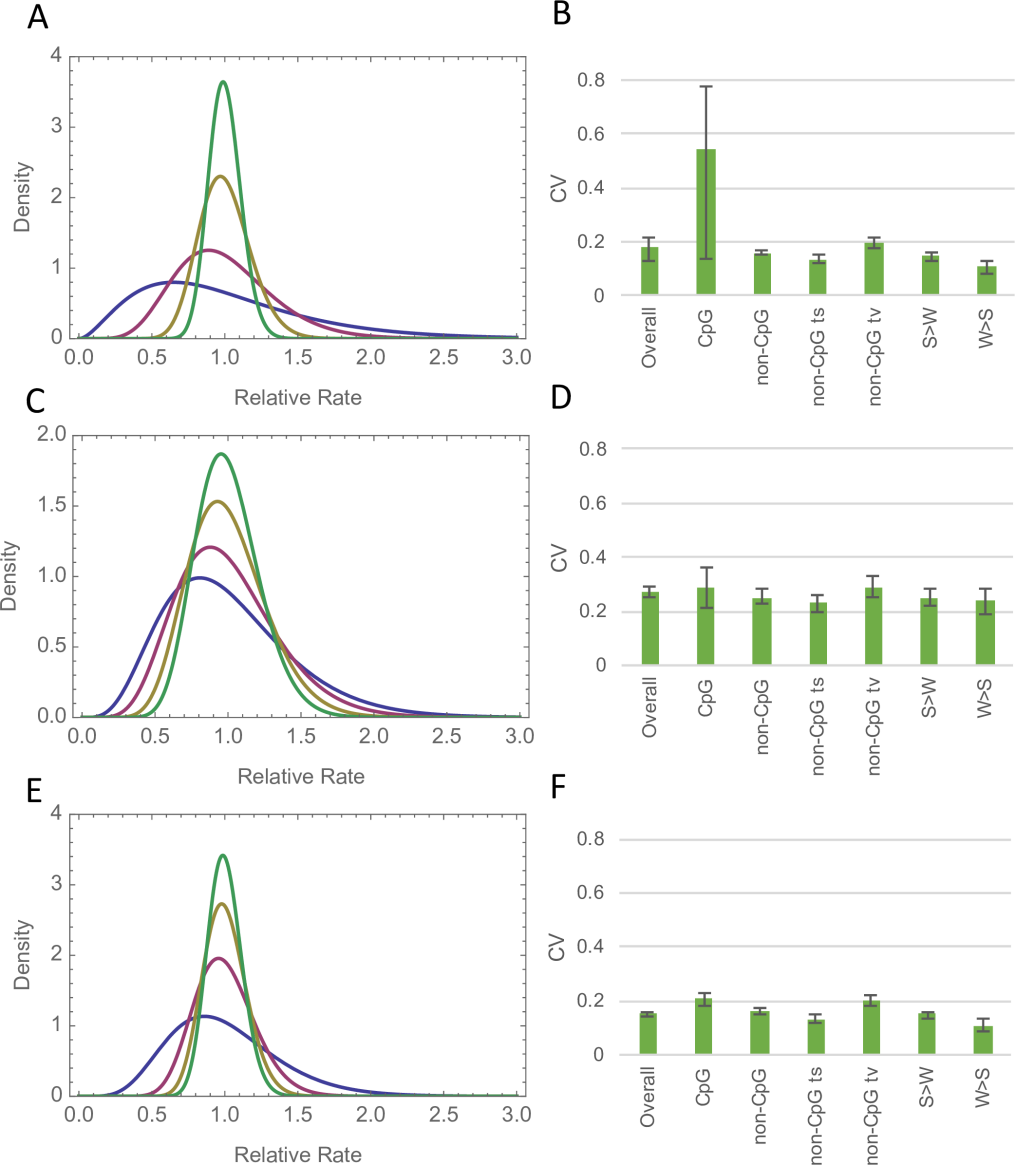
Gamma distribution fitted at different scales and to different categories of mutation. The gamma distribution fitted to the number of DNMs per window at different scales: 10KB (blue) 100KB (maroon), 1MB (olive) and 10MB (green) for the A) Francioli C) Wong, and E) Jonsson data; and the CV of the distribution fitted to various mutational categories at the 1MB scale for B) Francioli D) Wong, and F) Jonsson data.

**Fig 4:**
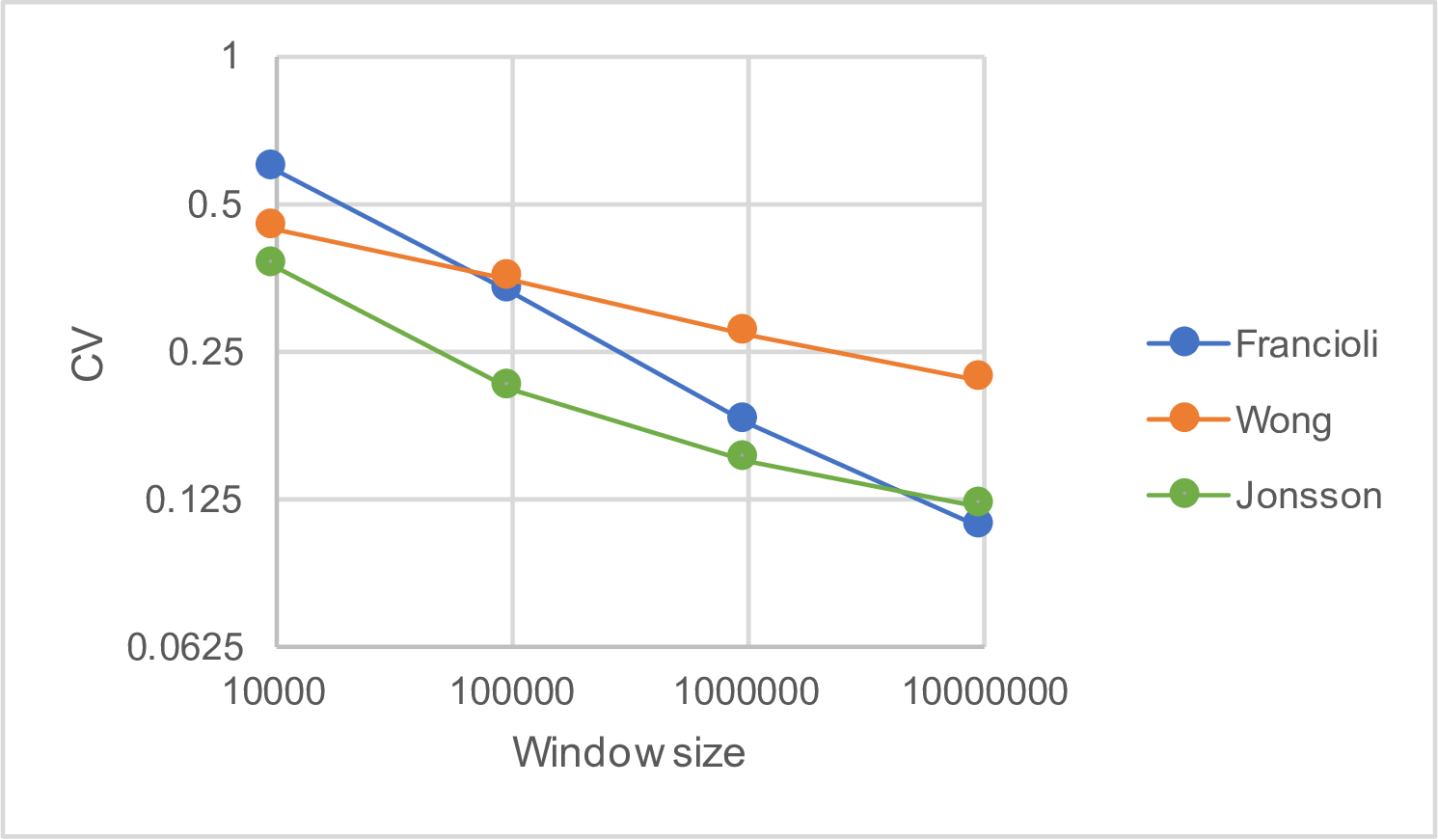
The coefficient of variation of the fitted distribution across scales. Note both variables are plotted on a log-scale.

If we include estimates of the distribution for 10KB, 100KB and 10MB we find, as expected, that the variance in the mutation rate declines as the scale gets larger (Figs 3 and 4). This is more marked for the Francioli dataset than for the Wong and Jonsson datasets (Figs 3 and 4). If we plot the CV of the fitted gamma distribution against the window size we find that the log of the CV of the gamma distribution is approximately linearly related to the log of the window size for the Francioli and Wong datasets (Fig 4); the relationship appears curvi-linear for the Jonsson dataset. The fact that the CV declines gradually across scales suggests that the variation at the 1MB scale is part of a continuum of variation at different scales. The linearity of the relationship in two of the datasets suggests that a simple phenomenon may underlie the variation at different scales.

If all the variation at the larger scales is explainable by variation at a smaller scale, then the CV at scale *x* should be equal to the CV at some finer scale, *y*, divided by the square-root of *x/y*; on a log-log scale this should yield a slope of -0.5. The slope for each dataset is shallower than this (Francioli b = -0.25; Wong b = -0.10; Jonsson b = -0.16). This therefore suggests that there is variation at a larger scale that cannot be explained by variation at a smaller scale. To test whether this is the case, we ran a series of one-way ANOVAs; testing variation at the 100KB scale using 10KB windows, 1MB using 100KB windows and 10MB using 1MB windows. The results were significant for all datasets (p<0.001 in all cases).

### Mutational types

If we estimate the distribution for individual mutational types we find that in many cases the lower CI on the CV is zero; this might be because we do not have enough data to reliably estimate the distribution for each individual mutational type. We therefore combined mutations into a variety of non-mutually exclusive categories. In each case we estimated the distribution for the relevant category of sites–e.g. in considering the distribution of CpG rates we consider the number of CpG DNMs at CpG sites, not at all sites. We find that the estimated distributions are similar for different mutational types except that there is rather more variation at CpG sites in the Francioli dataset (Fig 3; 100KB results S1 Table). Although the distributions are fairly similar for different mutational types, likelihood ratio tests demonstrate that there are significant differences between mutational categories (S2 Table for 1MB and 100KB results); this is particularly apparent for the Jonsson dataset, probably as a consequence of the size of this dataset. Never-the-less the differences between different mutational categories are relatively small.

### Correlations between mutational types

Given that there is variation in the mutation rate at the 1MB scale and that this variation is quite similar in magnitude for different mutational types, it would seem likely that the rate of mutation for the different mutational types are correlated. We find that this is indeed the case. We observe significant correlations between all categories of mutations in the three datasets (Table 1; S3 Table for 100KB). The correlations are weak but this is to be expected given the large level of sampling error. To compare the correlation to what we might expect if the two categories of mutation shared a common distribution and were perfectly correlated, we simulated data under a common distribution, estimating the CV of the common distribution as the mean of the distributions fitted to the two mutational categories. We find that generally the observed correlations are similar, and not significantly different, to the expected correlations. In some cases, we observe that the simulated correlation is actually consistently weaker than the observed correlation; this may reflect the inadequacy of the gamma distribution in describing the distribution of rates.

**Table 1:** The correlation between different mutational types at the 1MB scale.

Comparison	Observed correlation	Expected correlation	Proportion of simulated correlations > observed
*Francioli*			
CpG v. nonCpG	0.097***	0.057	0.96
nonCpG ts v. nonCpG tv	0.052**	0.036	0.73
S>W v. W>S	0.061**	0.024	0.94
*Wong*			
CpG v. nonCpG	0.16***	0.16	0.48
nonCpG ts v. nonCpG tv	0.22***	0.20	0.83
S>W v. W>S	0.22***	0.18	0.95
*Jonsson*			
CpG v. nonCpG	0.16***	0.26	0.0
nonCpG ts v. nonCpG tv	0.31	0.24	1.0
S>W v. W>S	0.23	0.17	1.0

The observed correlation is given along with the mean correlation from simulated data under the assumption that the two categories have the same distribution and are perfectly correlated. The proportion of 100 simulations in which the simulated correlation was less than the observed is also given

* p<0.05

**p<0.01

***p<0.001

### Variation in base composition

The fact that the rates of Strong to Weak base pairs (S>W) and W>S mutation covary (Table 1) suggests that mutational biases are unlikely to generate much variation in GC-content across the genome. To investigate this further, we used two approaches to test whether there was variation in the pattern of mutation that could generate variation in GC content. First, we used the DNM data for each window to predict the equilibrium GC content to which the sequence would evolve, fitting a model by maximum likelihood (ML) in which this equilibrium GC-content could vary across the genome. The ML estimate for the mean equilibrium GC-content is similar in all datasets at ~0.32. The ML estimate and its 95% CIs for the standard deviation for the equilibrium GC-content are 0.02 (0, 0.060), 0.001 (0, 0.036) and 0.011 (0, 0.024) for the Francioli, Wong and Jonsson respectively; in each case confidence intervals encompass 0, suggesting that a model with no variation in equilibrium GC-content fits the data well. Furthermore, the upper confidence interval is small, suggesting that at most variation in the pattern of mutation generates little variation in GC-content.

**Fig 5:**
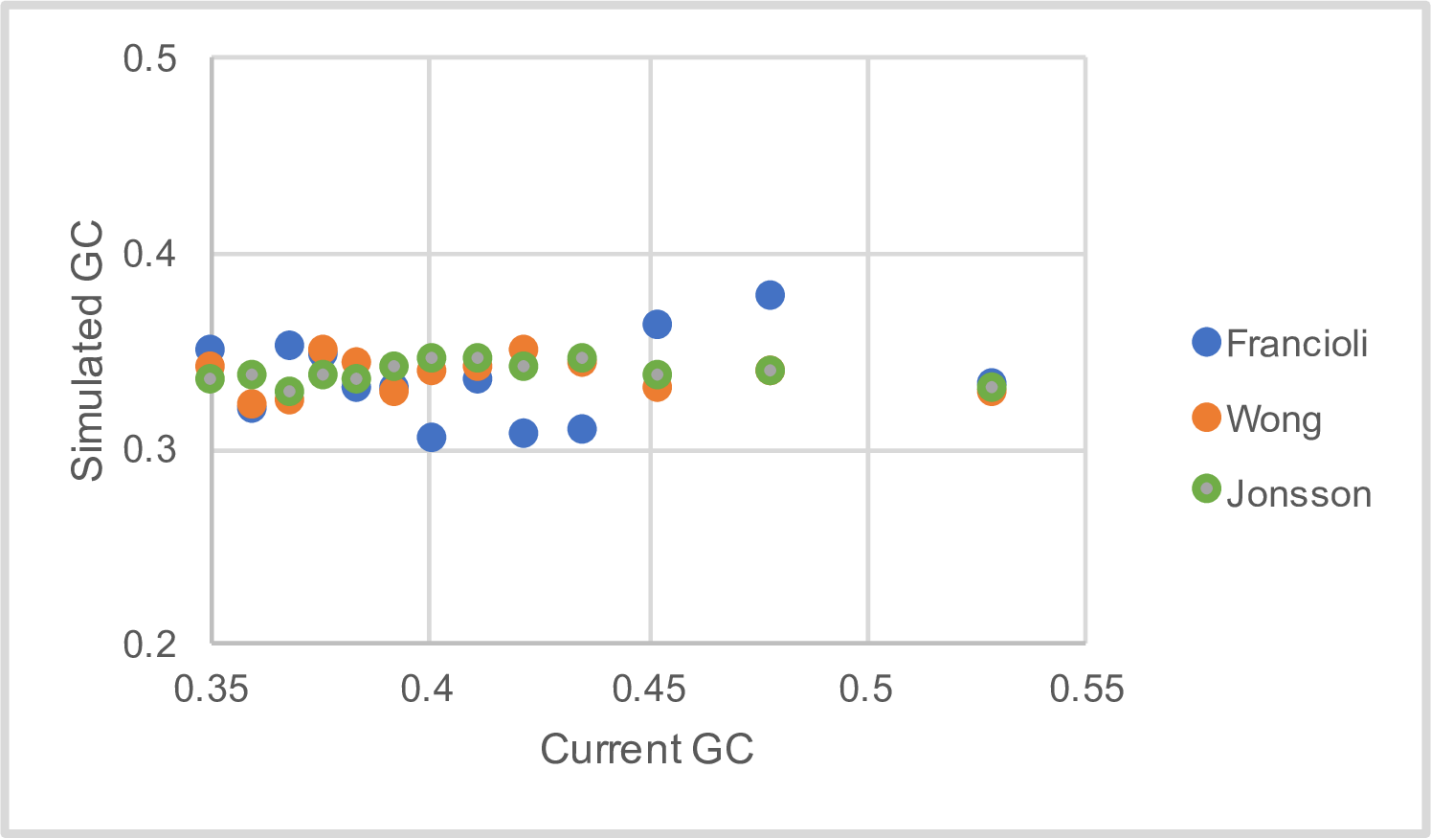
The equilibrium GC content from a simulation of sequence evolution. The equilibrium GC content from a simulation of sequence evolution is plotted against the current GC-content of the windows from which the mutation pattern was estimated. Note several of the points are coincident.

However, the ML method does not rule out the possibility that there is some variation in the pattern of mutation. Furthermore, the method does not take into account the difference in the mutation rate between CpG and non-CpG sites. We therefore used a second approach in which we grouped windows together based on their current GC-content. We then estimated the mutation rates for the 9 categories of mutation using the DNM data and used these estimated mutation rates in a simulation of sequence evolution, in which we evolved the sequence to its equilibrium GC content. We find no correlation between the equilibrium GC content to which the sequence evolves and the current GC content (Fig 5; S4 Fig for 100KB).

### Mutation models

It has been suggested that the mutation rate at a site is predictable based on genomic features, such as replication time, by Michaelson et al. [5], or the 7-mer sequence in which a site is found, by Aggarwala et al. [40]. To investigate whether these models can explain the variation at large scales we used the models to predict the average mutation rate for each 100KB or 1MB region and correlated these predictions against the observed number of DNMs per site.

We find that the density of DNMs is significantly correlated to the rates predicted under the 7-mer model of Aggarwala et al. [40]. This correlation is significantly positive for the Wong and Jonsson datasets, as we might expect, but significantly negative for the Francioli dataset (Table 2; S4 Table for 100KB results). To compare these correlations to what we might expect if the Aggarwala model explained all the variation at large scales, we simulated the appropriate number of DNMs across the genome according to this model. The observed correlation is significantly smaller than the expected correlation for all datasets, however, the observed and expected correlations are quite similar for the Wong dataset suggesting that much of the variation in DNM density in this dataset is explainable by the model of Aggarwala et al. [40]. However, the model explains almost none of the variation in the Jonsson dataset.

**Table 2:** Correlation between the density of DNMs and the mutation rate estimates from the models of Aggarwala et al. [40] and Michaelson et al. [5] at the 1MB scale.

	Aggarwala		Michaelson	
	Observed	Expected	Observed	Expected
Francioli	-0.16***	0.16***	0.084***	0.41***
Wong	0.18***	0.25***	0.018	0.58***
Jonsson	0.068***	0.44***	0.13***	0.80***

The expected values are the mean correlations observed from 1000 simulations

*** p < 0.001

In contrast, the density of DNMs is significantly positively correlated to the predictions of the Michaelson model in the Francioli and Jonsson datasets, but not for the Wong dataset. However, in all cases the correlation is substantially and significantly smaller than it could be if the model explained all the variation (Table 2; S4 Table for 100KB results) suggesting that this model fails to capture much of the variation at the 1MB and 100KB scales.

### Correlations with genomic variables

To try and understand why there is large scale variation in the mutation rate, we compiled a number of genomic variables which have previously been shown to correlate to the rate of germline or somatic DNM, or divergence between species: male and female recombination rate, GC content, replication time, nucleosome occupancy, transcription level, DNA hypersensitivity and several histone methylation and acetylation marks [3, 5, 9, 41, 42].

Surprisingly, the three datasets yield different patterns of correlation. The overall density of DNMs is significantly positively correlated to male and female recombination rates across all datasets, but otherwise there is no consistency (Table 3; 100KB results S5 Table); for example, DNM density is negatively correlated to replication time (later replicating regions have higher mutation rates) in the Francioli and Jonsson datasets, but positively correlated in the Wong dataset, and despite containing 10-times as much data, the correlation is weaker in the Jonsson than the Francioli dataset. Overall, the correlations are more similar in their direction in the Francioli and Jonsson datasets.

**Table 3:** The correlation between the density of DNMs and various genomic variables at the 1MB scale.

	Francioli	Wong	Jonsson	Wong (callable)	W<>W and S<>S substitutions
Male recombination rate	0.072***	0.225***	0.156***	0.208***	0.254***
Female recombination rate	0.06***	0.240***	0.084***	0.215***	0.116***
H3K4me1	-0.097***	0.156***	-0.002	0.123***	-0.136***
H3K4me3	-0.176***	-0.012	-0.066**	-0.032	-0.424***
H3K27me3	-0.080***	0.039	-0.025	0.027	-0.199***
H3K27ac	-0.134***	0.10***	-0.019	0.070**	-0.396***
Transcription rate	-0.122***	-0.033	-0.003	-0.043*	-0.214***
H3K4me1PB	-0.119***	0.105***	-0.062**	0.080***	-0.385***
H3K9me3PB	0.106***	-0.169***	0.012	-0.133***	0.420***
Nucleosome occupancy	-0.070**	0.224***	0.019	0.184***	-0.357***
DNAse hypersensitivity	-0.144***	0.086***	0.013	0.062**	-0.302***
Replication time	-0.154***	0.045*	-0.087***	0.019	-0.474***
GC content	-0.110***	0.167***	0.032	0.132***	-0.324***

Also shown are the correlations when the number of DNMs in the Wong dataset divided by the sum of the callable trios and the number of W<>W and S<>S substitutions per site between human and chimpanzee.

* p<0.05

**p<0.01

***p<0.001.

Many of the genomic variables are correlated to each other. If we use principle components to reduce the dimensionality, the first principle component (PC) explains 58% of the variation in the genomic variables, the second 13%, the third and fourth 6.9 and 5.7% of the variation. We find that the density of DNMs is significantly negatively correlated to the first PC in the Francioli data (r = -0.14, p<0.001), significantly positively in the Wong data (r = 0.14, p<0.001) and uncorrelated in the Jonsson data (r = -0.013, p = 0.54). All are significantly positively correlated to the second PC (Francioli, r = 0.14, p<0.001; Wong, r = 0.27, p<0.001; Jonsson, r = 0.15, p < 0.001), uncorrelated to the third component and Wong and Jonsson are significantly correlated to the fourth component but in opposite directions (Wong, r = -0.059, p = 0.005; Jonsson, r = 0.1, p<0.001).

It is possible that the differences between Wong and the other datasets are due to biases in the ability to call DNMs. However, analysing the Wong data using the number of callable trios at each site does not qualitatively alter the pattern of correlation in the Wong dataset (Table 3) or the correlations to the principle components of the genomic features (PC1, r = 0.11 p<0.001; PC2, r = 0.25, p<0.001; PC3, r = -0.019, p = 0.37; PC4, r = -0.048, p = 0.019).

To investigate whether these patterns are consistent across mutational types, we calculated the correlation between the density of each mutational type (e.g. CpG C>T mutations at CpG sites) and the first two PCs of the genomic features. For the Francioli and Jonsson datasets the patterns are perfectly consistent; all mutational types, if they show a significant correlation, are significantly negatively correlated to the first PC, and significantly positively correlated to the second (S6 Table). For the Wong data, the patterns are more heterogeneous; all mutational types are positively correlated to the second PC, but some mutational types are significantly positively correlated to the first PC and others significantly negatively correlated.

In order to try and disentangle which factors might be most important in determining the rate of mutation we used stepwise regression. We find, as expected, that the models selected for the three datasets are different (Table 4); only male recombination rate is common to and correlated in the same direction in all three models. The differences are not due to variation in the ability to call DNMs in the Wong dataset since repeating the analyses using the sum of callable trios rather than sites, does not alter the patterns (Table 4). At the 100KB scale, replication time joins male recombination factor as a common factor in all three datasets (S7 Table).

**Table 4:** The standardised regression coefficients from a stepwise multiple regression with forward variable selection.

	Francioli	Wong	Jonsson	Wong (callable)
Male recombination rate		0.10***	0.14***	0.10***
Female recombination rate	0.069**	0.091**		0.084**
H3K4me1	0.12**			
H3K4me3	-0.084*	-0.13***		-0.13***
H3K27me3				
H3K27ac				
Transcription rate				
H3K4me1PB				
H3K9me3PB			-0.074*	
Nucleosome occupancy		0.49***	-0.12*	0.44***
DNAse hypersensitivity	-0.12**	0.14*		0.15*
Replication time	-0.12**		-0.12*	
GC content		-0.39**	0.24**	-0.38**
r^2^	0.044	0.10	0.042	0.084

Parameters had to be significant at p<0.05 to be added to the model.

* p <0.05

** p < 0.01

*** p < 0.001

The differences between the three datasets could be due to paternal age since Francioli et al. [3] showed that the correlation between DNM density and replication time was only evident amongst individuals born to young fathers (<28 years), and paternal age differs between the three studies: the average paternal age was 27.7 years in the Francioli dataset (Laurent Francioli pers comm), 33.4 years in the Wong data [6] and 32.0 in the Jonsson data (calculated from their supplementary data). To investigate whether this could explain the differences between the datasets we divided the DNMs into those discovered in individuals with young (<28 years) and old fathers (≥28 years), and regressed the normalised DNM density (dividing by the mean DNM density for each dataset in each age cohort) against replication time and PC1. We find no evidence that the relationship between DNM density and replication time (or PC1) is stronger in individuals born to young fathers in the Wong and Jonsson datasets (Table 5).

**Table 5:** Testing for an effect of paternal age.

	Replication time			PC1		
	Young	Old	P-value	Young	Old	P-value
Wong	2.81 (1.29)*	0.839 (0.502)	n.s.	0.0577 (0.0202)**	0.0500 (0.0078)***	n.s.
Jonsson	-0.656 (0.429)	-1.23 (0.30)***	n.s.	-0.00484 (0.00673)	-0.00148 (0.00467)	n.s.

The slope of the regression, and its standard error, between DNM density and replication time or PC1 in individuals born to young (less than 28 years) or old (28 years and older) fathers. The DNM density was normalised for each age group such that the mean DNM density was one. Also given is the p-value from a test of whether the slopes are the same.

* p<0.05

** p<0.01

*** p<0.001.

The amount of variation explained by the multiple regression models is small– 0.044, 0.10 and 0.042 for Francioli, Wong and Jonsson respectively—but this might be expected given the small number of DNMs per MB and hence the large sampling error. To investigate how much of the explainable variance the model explains we sampled rates from the gamma distribution fitted to the distribution of DNMs across the genome and generated DNMs using these rates and then correlated these simulated rates to the true rates (i.e. those sampled from the gamma distribution). The average coefficient of determination for the simulated data is 0.11, 0.39 and 0.42 for the Francioli, Wong and Jonsson datasets respectively suggesting that the regression model explains ~37%, ~26% and ~10% of the explainable variance for the three datasets. In all cases, none of the simulated datasets have a coefficient of determination that is as low as the observed.

### Correlation with divergence

The rate of divergence between species is expected to depend, at least in part, on the rate of mutation. To investigate whether variation in the rate of substitution is correlated to variation in the rate of mutation we calculated the divergence between humans and chimpanzees, initially by simply counting the numbers of differences between the two species. There are at least three different sets of human-chimpanzee alignments: pairwise alignments between human and chimpanzee (PW)[43] found on the University of California Santa Cruz (UCSC) Genome Browser, the human-chimp alignment from the multiple alignment of 46 mammals (MZ)[44] from the same location, and the human-chimp alignment from the Ensembl Enredo, Pecan and Ortheus primate multiple alignment (EPO) [45].

**Fig 6:**
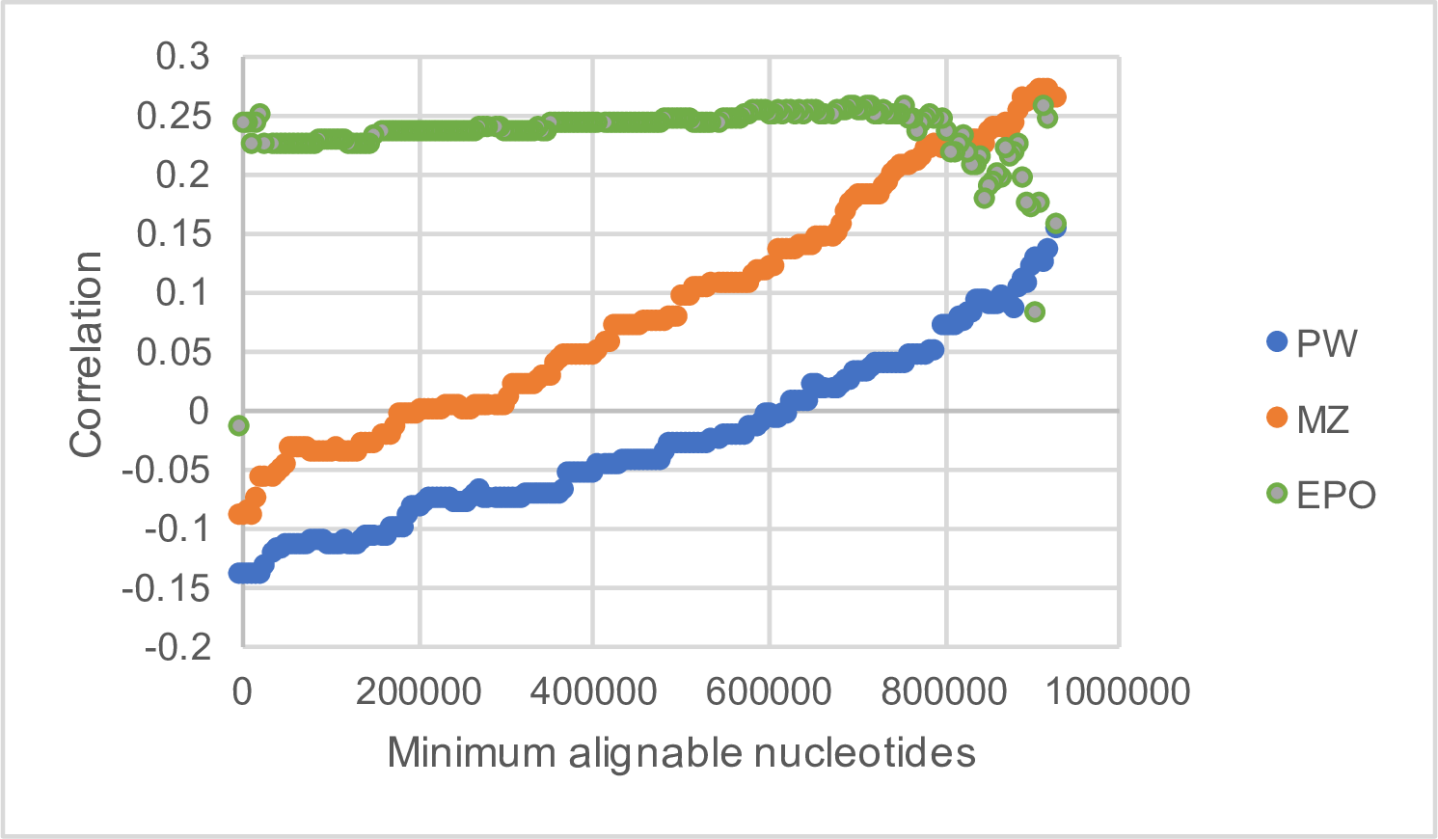
The quality of human-chimp alignments. The correlation between the divergence from human to chimpanzee and the density of DNMs in humans is plotted against the number of aligned sites per 1MB window for three sets of alignments: UCSC pairwise alignments (PW, blue), UCSC multi-way alignments (MZ, orange) and EPO multi-species alignments (EPO, green).

We find that the correlation depends upon the human-chimpanzee alignments used and the amount of each 1MB window covered by aligned bases (Fig 6). The correlation is significantly negative if we include all windows for the UCSC PW and MZ alignments at the 1MB scale, but becomes more positive as we restrict the analysis to windows with more aligned bases. In contrast, the correlations are always positive when using the EPO alignments, and the strength of this correlation does not change once we get above 200,000 aligned bases per 1MB. Further analysis suggests there are some problems with the PW and MZ alignments because divergence per MB window is negatively correlated to mean alignment length (r = -0.31, p < 0.0001) for the PW alignments and positively correlated (r = 0.57, p < 0.0001) for the MZ alignments (S5 Fig). The EPO alignment method shows no such bias and we consider these alignments to be the best of those available. Therefore, we use the EPO alignments for the rest of this analysis.

To gain a more precise estimation of the number of substitutions we used the method of Duret and Arndt [21], which is a non-stationary model of nucleotide substitution that allows the rate of transition at CpG dinucleotides to differ to than that at other sites. As expected the divergence along the human lineage (since humans split from chimpanzees) is significantly correlated to the rate of DNMs (Francioli, r = 0.20 p<0.001; Wong, r = 0.16, p<0.001; Jonsson, r = 0.31, p<0.001). However, the correlation between the rate of DNMs and divergence is not expected to be perfect even if variation in the mutation rate is the only factor affecting the rate of substitution between species; this is because we have relatively few DNMs and hence our estimate of the density of DNMs is subject to a large amount of sampling error. To investigate how strong the correlation could be, we follow the procedure suggested by Francioli et al. [3]; we assume that variation in the mutation rate is the only factor affecting the variation in the substitution rate across the genome between species and that we know the substitution rate without error (this is an approximation, but the sampling error associated with the substitution rate is small relative to the sampling error associated with DNM density because we have so many substitutions). We generated the observed number DNMs according to the rates of substitution, and then considered the correlation between these simulated DNM densities and the observed substitution rates. We repeated this procedure 1000 times to generate a distribution of expected correlations. Performing this simulation, we find that we would expect the correlation between divergence and DNM density to be 0.30, 0.44 and 0.68 for the Francioli, Wong and Jonsson datasets respectively, considerably greater than the observed values of 0.20, 0.16 and 0.31 respectively. In none of the simulations was the simulated correlation as low as the observed correlation.

There are several potential explanations for why the correlation is weaker than it could be; the pattern of mutation might have changed [39, 46–48], or there might be other factors that affect divergence. Francioli et al. [3] showed that including recombination in a regression model between divergence and DNM density significantly improved the fit of the model; a result we confirm here; the coefficient of determination when the sex-average recombination rate is included in a regression of divergence versus DNM density increases from 0.039 to 0.14, 0.026 to 0.12 and 0.095 to 0.18 for the Francioli, Wong and Jonsson datasets respectively; similar patterns are observed for male and female recombination rates separately.

As detailed in the introduction there are at least four explanations for why recombination might be correlated to the rate of divergence independent of its effect on the rate of DNM: (i) biased gene conversion, (ii) recombination affecting the efficiency of selection, (iii) recombination affecting the depth of the genealogy in the human-chimpanzee ancestor and (iv) problems with regressing against correlated variables that are subject to sampling error. We can potentially differentiate between these four explanations by comparing the slope of the regression between the rate of substitution and the recombination rate (RR), and the rate of DNM and the RR. If recombination affects the substitution rate, independent of its effects on DNM mutations, because of GC-biased gene conversion (gBGC), then we expect the slope between divergence and RR to be greater than the slope between DNM density and RR for Weak>Strong (W>S), smaller for S>W, and unaffected for S<>S and W<>W changes. The reason is as follows; gBGC increases the probability that a W>S mutation will get fixed but decreases the probability that a S>W mutation will get fixed. This means that regions of the genome with high rates of recombination will tend to have higher substitution rates of W>S mutations than regions with low rates of recombination hence increasing the slope of the relationship between divergence and recombination rate. The opposite is true for S>W mutations, and S<>S and W<>W mutations should be unaffected by gBGC. If selection is the reason that divergence is correlated to recombination independently of its effects on the mutation rate, then we expect all the slopes associated with substitutions to be less than those associated with DNMs. The reason is as follows; if a proportion of mutations are slightly deleterious then those will have a greater chance of being fixed in regions of low recombination than high recombination. If the effect of recombination on the substitution rate is due to variation in the coalescence time in the human-chimp ancestor, then we expect all the slopes associated with substitution to be greater than those associated with DNMs; this is because the average time to coalescence is expected to be shorter in regions of low recombination than in regions of high recombination. Finally, if the effect is due to problems with multiple regression then we might expect all the slopes to become shallower. Since the DNM density and divergences are on different scales we divided each by their mean to normalise them and hence make the slopes comparable.

**Fig 7:**
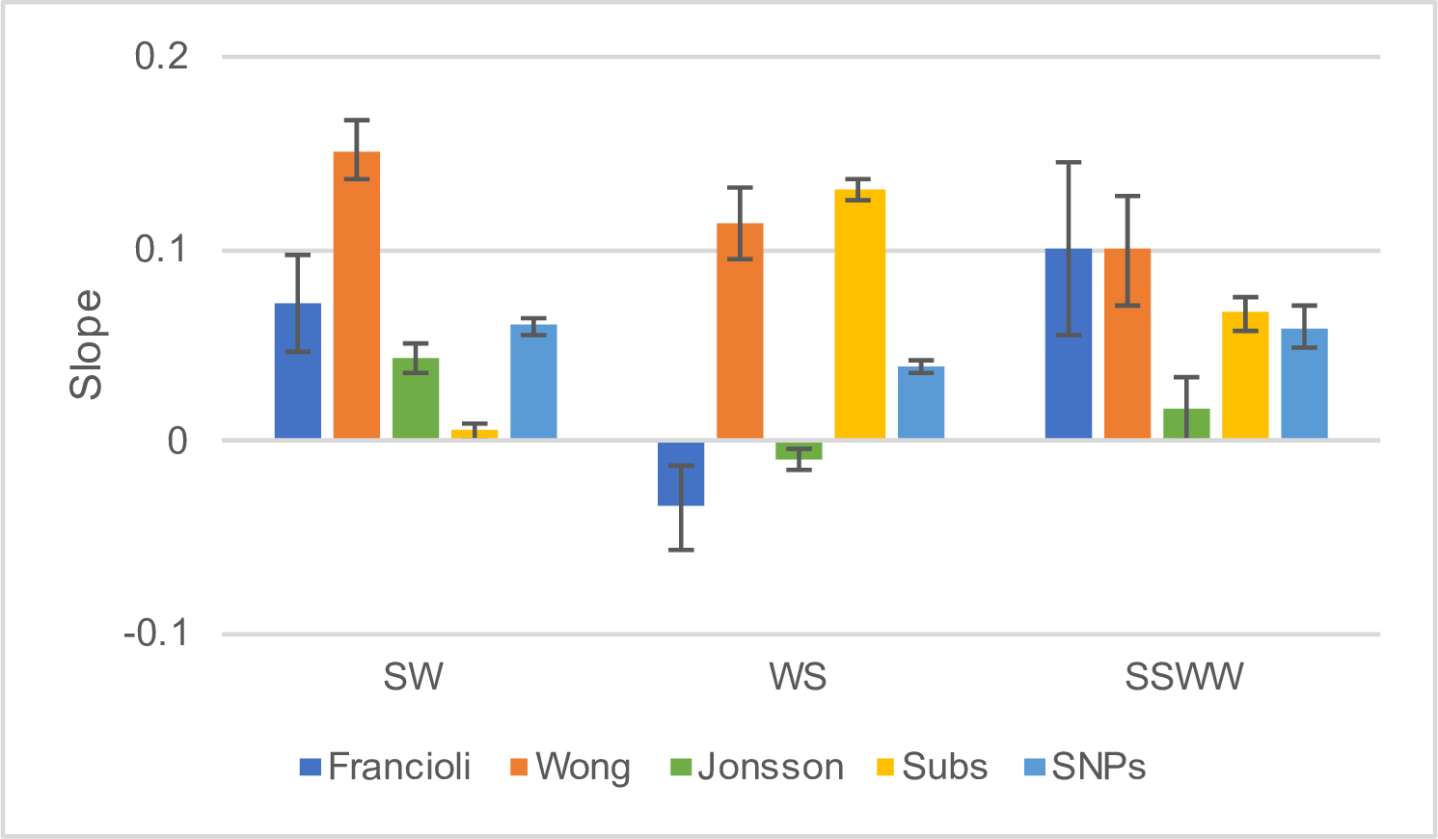
Testing why divergence is correlated to recombination rate. The slope (and SE) between normalised DNM density and normalised sex-averaged recombination rate (RR) (Wong—blue, Francioli–orange, Jonsson–green), normalised substitution density and RR (yellow) and normalised SNP density and RR (light blue). In each case the values were normalised by dividing by the mean.

The results of our test are consistent with the gBGC hypothesis; the slope of divergence versus RR is greater than the slope for DNM density versus RR for W>S mutations and less for S>W mutations (Fig 7); we present the analyses using sex-averaged RR, but the results are similar for either male or female recombination rates, and for 100KB windows (S6 and S7 Figs and S8 and S9 Tables). These differences are significant in the expected direction for all comparisons except W>S from the Wong data (Table 6)(significance was assessed by bootstrapping the data by MB regions 100 times and then recalculating the slopes). There are no significant differences between the slope for W<>W and S<>S mutations and the slope for substitutions, consistent with gBGC, except for the Jonsson dataset in which the DNM slope is significantly less than the slope for substitutions. This latter result suggests that there might also be an effect of linked selection, but this result should be treated with caution given that the other two datasets show the opposite pattern.

**Table 6:** Testing the difference in slopes.

	S>W	W>S	S<>S W<>W
*Substitutions*			
Francioli	0.99	0	0.74
Wong	1	0.13	0
Jonsson	1	0	0
*SNPs*			
Francioli	0.8	0	0.83
Wong	1	1	0.95
Jonsson	0	0	0

Given is the proportion of bootstrap replicates (out of 100) in which the slope of the normalised DNM density versus sex-averaged recombination rate, is greater than the slope of the normalised number of substitutions (or SNPs) versus recombination rate.

### Correlation with diversity

Just as we expect there to be correlation between divergence and DNM rate, so we might expect there to be correlation between DNA sequence diversity within the human species and the rate of DNM. To investigate this, we compiled the number of SNPs in 1MB and 100KB windows from the 1000 genome project [49, 50]. There is a positive correlation between SNP density and DNM density in all datasets (Francioli r = 0.18 p<0.001; Wong r = 0.31, p<0.001; Jonsson r = 0.43, p<0.001).

Using a similar strategy to that used in the analysis of divergence we calculated the correlation we would expect if all the variation in diversity was due to variation in the mutation rate by assuming that the level of diversity is known without error, and hence is a perfect measure of the mutation rate (we have on average 31,000 SNPs per MB, so there is little sampling error associated with the SNPs). We then simulated the observed number of DNMs according to these inferred mutation rates. The expected correlations are 0.24, 0.35 and 0.58 in the Francioli, Wong and Jonsson datasets, which are slightly higher than the observed correlation, significantly so for Francioli and Jonsson (p<0.01 in both cases). The observed correlations are 74%, 89% and 74% of the expected correlations for Francioli, Wong and Jonsson respectively. A similar pattern is observed for individual mutational types at both the 1MB and 100KB scale, with some being greater and others smaller than expected (S10 Table). These results suggest that much of the variation in diversity at the 1MB scale is due to variation in the mutation rate.

Although much of the variation in diversity appears to be due to variation in the mutation rate we tested for the effect of gBGC. We find the slopes are consistent with gBGC for the Francioli dataset, but the other datasets show inconsistent patterns; in the Wong data, the slope of DNM versus RR is significantly greater than the slope of SNP density versus RR across all mutational categories and the opposite pattern is found in Jonsson (p<0.01 in all cases) (Fig 7).

### Divergence to other species

**Fig 8:**
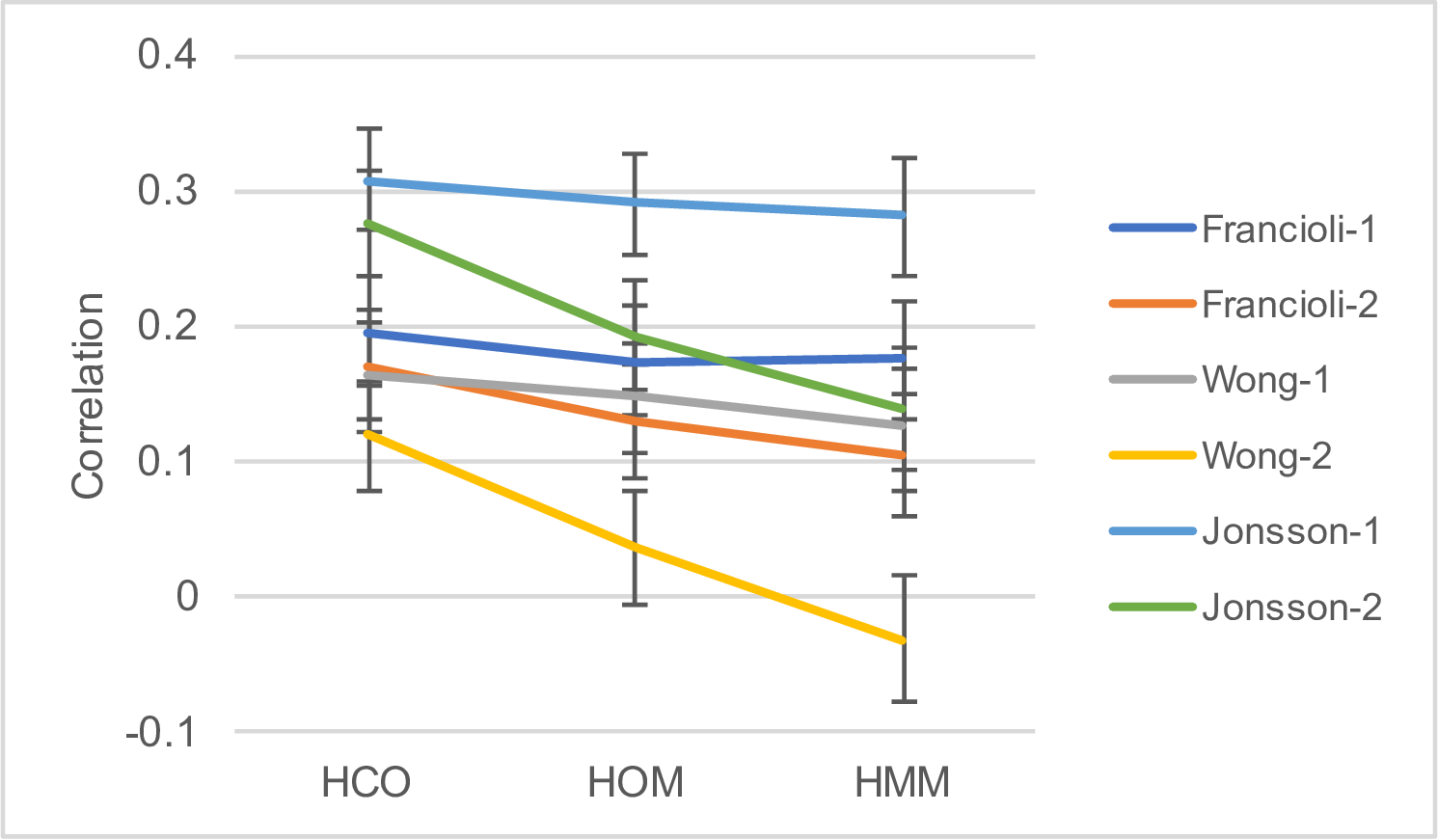
The decrease in the correlation between DNM density and divergence with increasing evolutionary divergence. The graph shows the correlation between DNM density and the substitution rate for different phylogenetic branches (with 95% confidence intervals). Francioli-1, Wong-1 and Jonsson-1 are the correlations involving the divergence along the human lineages from the comparison of human-chimp-orangutan (HCO), human-orangutan-macaque (HOM) and human-macaque-marmoset (HMM). Francioli-2, Wong-2 and Jonsson-2 involve the divergences along the chimpanzee, orangutan and macaque lineages.

The divergence between species, usually humans and macaques, is often used to control for mutation rate variation in various analyses. But how does the correlation between divergence and the DNM rate in humans change as the species being compared get further apart? Terekhanova et al. [48] showed that the rate of S<>S and W<>W substitutions (chosen to eliminate the influence of gBGC) along the human lineage at the 1MB scale is correlated to that along other primate lineages, but that the correlation declines as the evolutionary distance increases. This suggests that the mutation rate evolves at the 1MB relatively rapidly. However, they did not consider DNMs in detail. To investigate further, we compiled data from a variety of primate species–human/chimpanzee/orangutan (HCO) considering the divergence along the human and chimp lineages, human/orangutan/macaque (HOM) considering the divergence along the human and orangutan lineages, and human/macaque/marmoset (HMM) considering the divergence along the human and macaque lineages. This yields two series of divergences of increasing evolutionary divergence: the human lineage from HCO, HOM and HMM, and chimp from HCO, orangutan from HOM and macaque from HMM. We estimated the divergence using the non-stationary method of Duret and Arndt [21] that treats CpG sites separately. We do not restrict ourselves only to DNMs in the aligned regions but used all DNMs in each window. In this way, the average number of DNMs per window is independent of the evolutionary divergence. As expected, we find that the correlation between the density of DNM and the rate of substitution declines as the evolutionary divergence increases, except the correlation between the density of DNMs in the Francioli dataset and the divergence along the human lineage since the divergence from orangutan which is slightly lower than the correlation with divergence since humans split from macaques (Fig 8). It is also notable that the decrease in the correlation is quite modest in many cases.

## Discussion

We have considered the large-scale (1MB or 100KB) distribution of DNMs along the human genome using an analysis of 3 datasets obtained by the sequencing of trios (an individual and their parents). Unfortunately, there are significant differences between these datasets; most conspicuously they show different patterns of correlation to genomic variables. For example, the density of DNMs at the 1MB scale is significantly negatively correlated to the density of H3K4me1 epigenetic marks in the dataset of Francioli et al. [3], significantly positively correlated in Wong et al. [6] and uncorrelated in Jonsson et al. [36] despite this being by far the largest dataset. However, these correlations to genomic variables are weak, and explain only a small fraction of the explainable variance, and there are many commonalities between datasets, which likely represent true patterns. There appears to be rather little variation in the mutation rate at a large scale in all datasets. However, there is variation at a large scale that cannot be explained by variation at smaller scales, and large-scale variation forms part of a continuum of variation across different scales. Furthermore, the level of variation for different mutational types is similar and different mutational types covary together. There is no evidence that variation in the pattern of mutation generates variation in GC content that would underlie the maintenance of isochores. In all datasets, the correlations to genomic variables are weak and explain little of the explainable variance. We confirm that the correlation between the mutation rate, as measured by DNM density, and divergence, is not as strong as it could be across datasets, and demonstrate that this is in part due to BGC. In contrast, we find that variation in diversity at large scales is largely a consequence of variation in the mutation rate. Finally, we demonstrate that the correlation between the rate of DNM and the rate of substitution, declines as increasingly divergent species are considered.

It is possible that the differences between datasets are due to parental age, since Francioli et al. [3] found that the correlation between DNM density and replication time was only evident in individuals born to young fathers, and paternal age differs between our datasets. However, like Besenbacher et al. [51], we find no evidence that paternal age affects the relationship between the mutation rate and replication time or genomic variables, as summarised by the first principle component of the genomic variables, in either the Wong or Jonsson datasets.

It is also possible that the differences between the datasets are due to ethnicity, since it has been shown that the rate and pattern of mutation, at the single nucleotide scale, varies over short timescales, such that it can vary between human populations [39, 46, 47]; for example, the rate of TCC to TTC is elevated in Europeans [39, 46]. It has also been demonstrated that the mutation pattern evolves at larger scales. Terekhanova et al. [48] considered the correlation between the rate of S<>S and W<>W substitution along the human and other primate lineages at the 1MB scale. They showed that the strength of the correlation declines as more distant species are considered suggesting that the mutation rate evolves at this scale. However, the rate of decline was fairly slow, and human populations would not be predicted to show very different patterns from this analysis. Furthermore, it seems that the populations considered by the three studies were dominated by individuals from the same population, Europeans: Dutch in the study of Francioli et al. [3], Icelanders in Jonsson et al. [36] and mostly North American Europeans in Wong et al. [6] (see [52] for ethic details).

**Fig 9:**
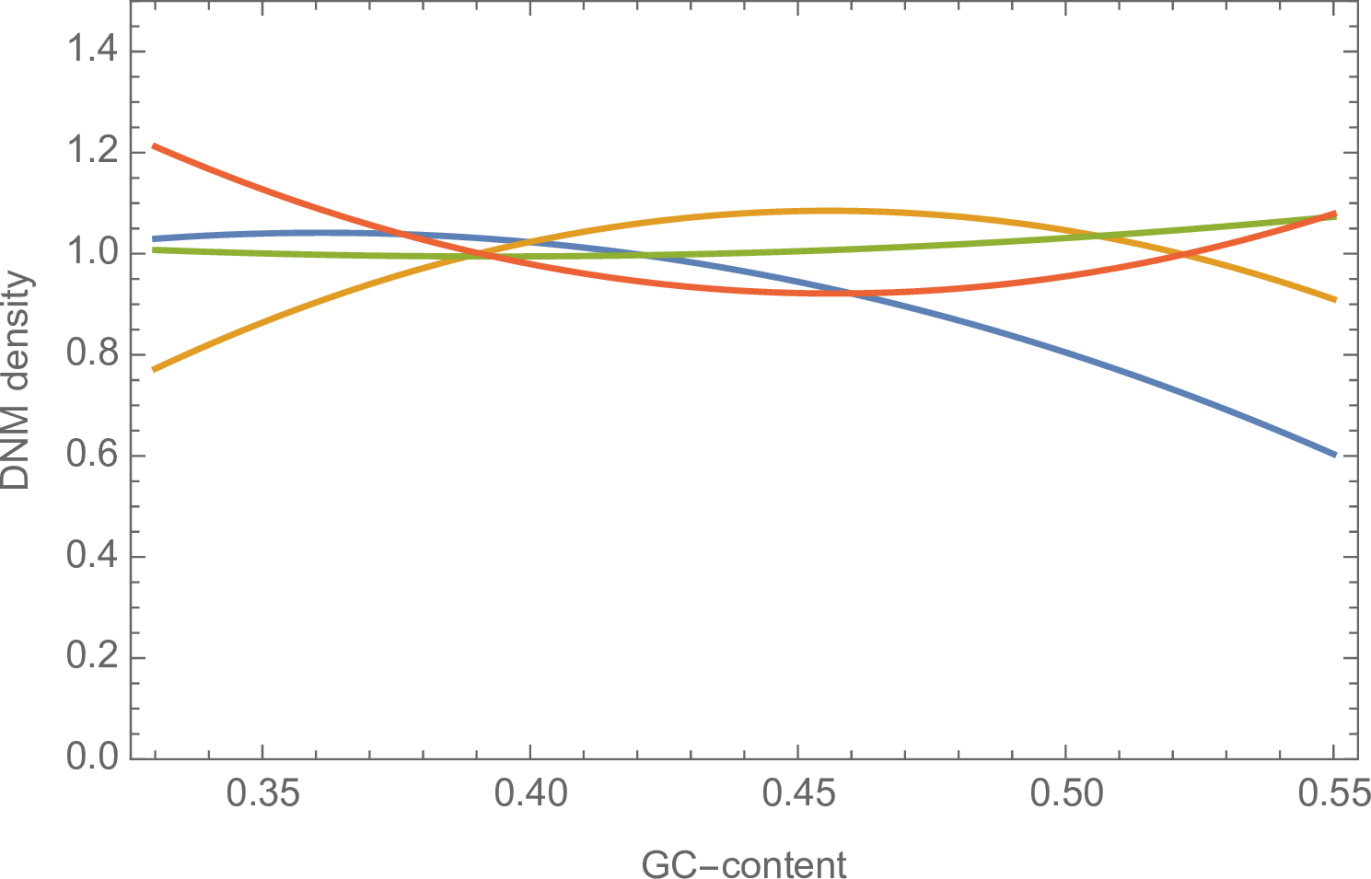
The relationship between DNM density, or divergence, and GC content. The relationship was estimated from a regression of DNM density or human-chimp divergence, against GC-content and the square of the GC-content at the 1MB scale. Blue–Francioli, Light Orange–Wong, Green–Jonsson and Dark Orange–W<>W and S<>S substitutions between human and chimpanzee.

Without any other obvious explanation, it therefore seems likely that the differences between datasets are due to sequencing technology, or the pipelines used to call the DNMs. The Francioli [3] and Jonsson [36] datasets were largely sequenced using Illumina Hiseq at 13x and 35x coverage respectively. The Wong [6] dataset was sequenced using the DNA nanoball technology at 60x coverage. The datasets were subject to a variety of different methods to call DNMs. One potential problem is a GC-bias that has been documented for Illumina sequencing [53], in which high and low GC-content reads are under-represented [54]. To investigate whether this might be the cause of the differences between datasets we regressed the number of DNMs per MB against GC content, and the square of the GC content, to allow for non-linearity. We find that both linear and quadratic terms are significant for the Francioli (p<0.05 for both terms) and Wong (p<0.001 for both terms) datasets, but neither coefficient is significant in the Jonsson dataset. In the Francioli dataset high GC-content regions have fewer DNMs, whereas in Wong it is the low GC-content regions that have a deficit (Fig 9). If we take the residuals from the regression and correlate these against genomic variables we find consistent patterns across datasets (Table 7): the GC-content corrected DNM density is significantly positively correlated to male and female recombination rates, and significantly negatively correlated to replication time and H3K4me3 across all datasets. There are some other significant correlations to histone marks in each of the datasets, with the sign of the correlation being consistent across datasets. If we calculate the principle components for the genomic variables, excluding GC-content, we find that the first four components explain 55, 14, 7.4 and 6.2% of the variance respectively. We find consistent patterns of correlation across datasets in terms of the sign of the correlation (Table 7)—the GC-corrected density of DNMs is negatively correlated to the first PC, but only significant for Jonsson, significantly positively correlated to the second PC in all datasets, uncorrelated to the third and only significantly correlated to the fourth in Jonsson (Table 7).

**Table 7:** Correlation between the GC-corrected density of DNMs and various genomic variables.

	Individual correlations			Multiple regression		
	Francioli	Wong	Jonsson	Francioli	Wong	Jonsson
Male recombination rate	0.092***	0.18***	0.15***		0.099***	0.15***
Female recombination rate	0.10***	0.16***	0.074***	0.078**	0.072**	
H3K4me1	-0.006	0.015	-0.028	0.13**	0.12**	
H3K4me3	-0.096***	-0.12***	-0.090**	-0.091**	-0.18***	
H3K27me3	-0.040	-0.023	-0.036			
H3K27ac	-0.047*	-0.037	-0.044*			0.11**
Transcription rate	-0.050*	-0.094***	-0.029			
H3K4me1PB	-0.051*	-0.015	-0.080***		0.055*	-0.071*
H3K9me3PB	0.022	0.004	0.032			
Nucleosome occupancy	0.020	0.040	-0.001			
DNAse hypersensitivity	-0.031	-0.024	-0.026			
Replication time	-0.090***	-0.079***	-0.10***	-0.13***	-0.087*	-0.14**
PC1	-0.040	-0.026	-0.048*			
PC2	0.14***	0.23***	0.15***			
PC3	-0.012	-0.010	0.000			
PC4	0.025	-0.005	0.084***			

Also given are standardised slopes from a multiple regression with forward parameter selection (p<0.05 for inclusion). The density of DNMs was corrected for GC-content by regressing DNM density against GC-content and the squared GC-content and taking the residuals.

* p < 0.05

** p < 0.01

*** p < 0.001

Despite the fact that the GC-corrected densities of DNMs show similar correlations to genomic variables, we do not find similar models selected by forward selection in a multiple regression (Table 7). Only one feature is common to all datasets–replication time. The differences between the datasets may reflect the strong correlations between genomic variables, which makes it difficult for any procedure to select the correct model.

The fact that correcting for GC content yields similar patterns of correlation to genomic variables, suggests that there is a GC-bias in detecting DNMs. However, regressing against GC-content does not necessarily yield the correct pattern, because there may be a genuine relationship between the mutation rate and GC-content. For example, if we regress the number of W<>W and S<>S substitutions, chosen to remove the influence of BGC, between human and chimpanzee against GC-content we find a U-shaped relationship, unlike that seen for any of the DNM datasets (Fig 9); this might reflect the true pattern. Are there any clues as to which dataset reflects the true pattern of correlation? If we consider the correlation between S<>S and W<>W substitutions, and genomic variables (1MB Table 3; 100KB S5 Table) we find the correlations most closely parallel those in the Francioli dataset; when there is a significant correlation both the substitution date and the Francioli DNMs are significant in the same direction. In contrast, the sign of the correlation is usually the same in the substitution and Jonsson datasets, but the correlations are often non-significant in the Jonsson data. Wong shows very different patterns with some significant correlations in opposite directions.

If the differences between datasets are due to sequencing and processing technology then this has important implications for understanding the reasons the mutation rate varies across the genome because no two datasets show identical patterns of correlation between DNM density and genomic variables. We would suggest that unless a pattern can be shown to be consistent across datasets generated by different sequencing and processing technologies then it must be treated with some caution.

There are two additional points to make about correlations to genomic variables. First, it is evident that many genomic variables are highly correlated to each other so disentangling them will be difficult. Applying multiple regression may not be informative because few of the genomic variables are known without error, so the variables which come out as correlated may not be the causative factors, but those known with the least error. Second, genomic variables explain rather little of the variance in the rate of DNM. This may be because the genomic variables are measured with considerable error, or it may be that we are not assaying the factors which are important; but what these might be, is far from clear.

The evolution of the large-scale variation in GC-content across the human genome has been the subject of much debate [25]; mutation bias [14–18], selection [13, 19, 20] and biased gene conversion [21–24] have all been proposed as explanations. There is good evidence that biased gene conversion has some effect on the base composition of the human genome [24, 26–28]. However, this does not preclude a role for mutation bias. We have tested the mutation bias hypothesis using the DNM data and two different tests. We find no evidence that the pattern of mutation varies across the genome in a way that would generate variation in GC-content. Instead we provide additional evidence that biased gene conversion influences the chance that mutations become fixed in the genome.

We find that previous models of mutation rate variation do not explain the variation in DNM density seen in our datasets at large scales. This is perhaps not surprising. The model of Michaelson et al. [5] was derived by regressing a small number (~600) of DNMs against a suite of genomic variables at multiple scales. So, whilst the model took into account genomic variables at large scales it was principally aimed at estimating the rate of mutation at a single site. The model of Aggarwala et al. [40] estimated the mutation rate at individual sites based on the 7-mer context. It therefore contained no explicit information about large-scale variation.

As expected the rate of divergence between species is correlated to the rate of DNM, however, the strength and even the sign of the correlation depends on the alignments being used. The correlations between divergence and DNM density are actually negative if no filtering is applied to the UCSC alignments, and there is a negative correlation between divergence and alignment length for the pairwise alignments from the UCSC genome browser, and a positive correlation for the multi-species alignment. It is clear that there are problems with these alignments and that they should be used with caution.

As Francioli et al. [3] showed, the correlation between divergence and DNM density is worse than it would be if variation in the mutation rate was the only factor affecting divergence. This is perhaps not surprising because the substitution rate depends both on the rate of mutation and the probability of fixation, both of which may vary across the genome. Francioli et al. [3] further demonstrated that although the rate of DNM is correlated to the rate of recombination, divergence is correlated to the rate of recombination independently of this effect. There are at least four explanations for the effect of recombination on divergence: (i) biased gene conversion, (ii) direct selection, (iii) linked selection and (iv) problems with multiple regression. We have provided evidence for an effect of biased gene conversion, but no clear evidence of three other factors–i.e. the slope of the regression between DNM density and RR is not significantly different to the slope of the regression between divergence and RR for S<>S and W<>W mutations, except in the Jonsson data. However, whilst the slope of DNMs versus RR is lower than the slope of divergence versus RR in Jonsson, we see the opposite pattern in the other two datasets.

The fact that there is no obvious effect of indirect selection is surprising given the results of McVicker et al. [29]. They showed that the divergence between humans and chimpanzees was significantly lower near exons and other regions of the genome subject to evolutionary constraint. A similar reduction was not observed in the divergence of human and macaque and human and dog, suggesting that the pattern was not due to selection outside exons, or regions identified as being subject to selection (though see Phung et al.[30] who detected a correlation between divergence and proximity to functional DNA in the divergence between humans and rodents). They therefore inferred that the reduction was due to the effect of linked selection reducing diversity in the human-chimpanzee ancestor. There are several possible reasons why we see no evidence of this effect in our analysis. First, our test may not be powerful enough. Second, the effects may be counteracted by direct selection which is expected to affect the slope of the regression between divergence and RR in the opposite direction to indirect selection. Third, the scale, magnitude and variation in the effects of indirect selection may be not large enough to affect the relationship between divergence and the rate of mutation; if there is little variation in the magnitude of the indirect effects of selection across the genome at the 1MB (or 100KB) level then indirect selection will have no effect on the correlation between the rate of mutation and divergence. McVicker et al. [29] and Phung et al.[30] presented evidence of indirect selection affecting the divergence between humans and chimpanzees, but over short scales of <100KB. It is possible that at fine scales indirect selection may be more important.

In contrast to the pattern with divergence, we find that much of the variation in diversity, at least at the 1MB and 100KB scales, can be explained by variation in the mutation rate. This suggests that much of the correlation between diversity and RR [31–33, 55–57] is due to variation in the mutation rate not to linked selection. However, the correlation between DNM density and diversity is not as strong as it could be and this could be due to linked selection. Considering that much of the variation in diversity is due to variation in the mutation rate, it is perhaps not surprising that the analysis of DNM density versus RR and SNP density versus RR slopes are inconclusive. The results from Francioli are consistent with BGC affecting the relationship between SNP and DNM density, but the data from Wong and Jonsson are not.

Divergence between species has often been used to control for mutation rate variation in humans (for example [29, 55, 58, 59]). This is clearly not satisfactory given that the correlation between divergence and rate of DNM is only about half as strong as it could and this correlation gets worse as more divergent species are considered (see also Terekhanova et al. [48]). Unfortunately, correcting for mutation rate variation is likely to be difficult because attempting to predict mutation rates from genomic features is unreliable, given that regression models explain less than half the explainable variation. Furthermore, the largest amounts of variation are at the smallest scales (Fig 4) where we have the lowest density of DNMs.

We find, as others have before [3, 5], that the rate of germ-line DNM is correlated to a number of genomic features. However, we find that these features explain less than 50% of the explainable variance leaving the majority of the variance unexplained. Our inability to predict the mutation rate might be because the genomic features have not been assayed in the relevant tissue, the germ-line, or that there are important features that have yet to be assayed. Interestingly, Terekhanova et al. [48] showed that this unexplained component of the substitution rate evolves more rapidly than the explained component. They demonstrated that the substitution rates at the 1MB level in a range of primate species were almost as well correlated to genomic features in humans, as the substitution rate along the human lineage. This implies that the variance in the substitution rate not explained by genomic features, evolves rapidly, given that the correlation between the substitution rate in humans and other lineages declines as they get more distant. There is clearly much we do not currently understand about the why there is large scale variation in the mutation rate and how it evolves through time. Understanding these patterns is challenging given that different datasets show different patterns. Never-the-less there are some patterns which are common to all datasets.

## Materials and methods

### DNM data

Details of DNM mutations were downloaded from the supplementary tables of the respective papers or from the relevant web-sites: 105,385 mutations from Jonsson et al. [36], 26,939 mutations from Wong et al. [6] and 11016 mutations from Francioli et al. [3]. The data from Jonsson et al. was mapped to hg38 so the liftover tool was used to map these to hg19. Only autosomal DNMs were used.

### Alignments

Three sets of alignments were used in this analysis, all based on human genome build hg19/GRCh37: (i) the University of California Santa Cruz (UCSC) pairwise (PW) alignments [43] for human-chimpanzee (hg19-panTro4 downloaded from http://hgdownload.cse.ucsc.edu/goldenpath/hg19/vsPanTro4/) (ii) the UCSC MultiZ (MZ) 46-way alignments [44] downloaded from http://hgdownload.cse.ucsc.edu/goldenpath/hg19/multiz46way/ and (iii) Ensembl Enredo, Pecan, Ortheus (EPO) 6 primate multiple alignment, release 74, [45] downloaded from ftp://ftp.ensembl.org/pub/release-74/emf/ensembl-compara/epo_6_primate/. We found that the EPO alignments were the most reliable–see main text–and they were used for the majority of the analyses.

### Selection and filtering of SNPs

All SNPs from the 1000 genomes project phase 3 [50] were downloaded from http://hgdownload.cse.ucsc.edu/gbdb/hg19/1000Genomes/phase3/. After removing all multi-allelic SNPs and, structural variants and indels we were left with 77,818,368 autosomal SNPs. After filtering out windows which had less than 50% of nucleotides aligning between human-chimpanzee-orangutan and no recombination rate scores we were left with 71,917,321 SNPs.

### Mutational models

We considered how well the variation at the 100KB and 1MB scale was predicted by two models of mutation rates: the rates estimated by Aggarwala et al. [40] based on the 7-mer context surrounding a site, and the rates estimated for each site by Michaelson et al. based on a variety of genomic features. The rates for Aggarwala et al. [40] were taken from their S7 Table, and the context of each site was used to predict the average mutation rate for each 100KB or 1MB window using their model. The mutability indices from the Michaelson et al. study [5] were provided by the authors. The analysis of the model of Michaelson et al. [5] is more complex since they give the probability of detecting a DNM in their data at each site in the genome, referred to as the mutability index (MI), but these do not translate directly into mutation rates. Using their DNM data we tabulated the number of sites in the genome with a given MI along with the number of DNMs from their study that had been observed at those sites. Because DNMs are not observed at some MIs we grouped MIs into groups of ten starting from the first MI with at least one DNM. We then regressed the log of the number of DNMs over the number of sites against the mean MI (see S8 Fig). The regression line was estimated to be log(mutation rate) = -6.73 + 0.0103 x MI. Using this equation, we predicted the mutation rate at each site in the genome. Michaelson et al. [5] give MIs mapped to hg18; we lifted these over the hg19 using the liftover tool.

### Genomic features

Male, female and sex-averaged standardised recombination rate data [60] were downloaded from http://www.decode.com/additional/male.rmap, which provides recombination rates in 10KB steps. For each 100KB and 1MB windows the recombination rate was calculated as the mean of these scores with a score assigned to the window in which the position of its first base resided. GC content was calculated directly from the human genome (hg19/GCRh37) for 100kb and 1Mb windows. All other feature data was taken from the ENCODE project [61] and downloaded from the UCSC genome browser. Where possible we used data from the embryonic stem cell line H1-hESC. The mean value was taken for each genome feature across the window. For replication time data, we downloaded the ENCODE Repli-seq wavelet smoothed signal data [62, 63], provided in 1KB steps, for the GM12878, HeLa, HUVEC, K562, MCF-7 and HepG2 cell lines. Replication times were assigned to windows based upon their start coordinates. We computed the mean replication time for all autosomes for 100KB and 1MB windows across all 6 cell lines. We measured transcription rate using RNA-seq data. Nucleosome occupancy was taken from the GM12878 cell line, histone modifications and RNA-seq data from the stem cell line H1-hESC. We only included windows in our analysis in which >50% of the window had data from all features.

### Statistical analysis

SPSS version 22 and *Mathematica* version 10 were used for all statistical analyses.

To estimate the mutation rate distribution we use the method of [8]. In brief, we assume that the mutation rate in each window is $\alpha \bar{u}$ where $\bar{u}$ is the average mutation rate per site and *α* is the rate above or below this mean. α is assumed to be gamma distributed. The number of mutations per window is assumed to be Poisson distributed with a mean $\alpha \bar{u}l$ where *l* is the length of the window. This means that the number of mutations per window is a negative binomial. In considering a particular category of mutations, such as CpG transitions, we considered the number of CpG transition DNMs at CpG sites. We fit the distribution using maximum likelihood using the *NMaximize* function in *Mathematica*. Initial analyses suggested that the maximum likelihood value of the mutation rate parameter was very close to the mean estimate of the mutation rate; as a consequence, to speed up the maximization we fixed the mutation rate to its estimated mean and found the ML estimate of the shape parameter of the gamma distribution.

We investigated the correlation between different types of mutation across windows by fitting a single distribution to both types of mutation, estimating the shape parameter of the shared distribution as the mean of the CV of the ML estimates of distributions fitted to the two categories independently. We then used this distribution to simulate data; we drew a random variate for each window from the distribution assigning this as the rate for that window. We then generated two Poisson variates with the appropriate means such that the total number of DNMs for each type of mutation was expected to be equal the total number of DNMs of those types.

To test whether the mutation pattern varied across the genome in a manner that would generate variation in the mutation rate we fit the following model. Let us assume that the mutation rate from strong (S) to weak (W) base pairs, where strong are G:C and weak are A:T, be *μ*(1 − *f_e_*), where μ is the mutation rate and *f*_*e*_ is the equilibrium GC-content to which the sequence would evolve if there was no selection or biased gene conversion. Let the mutation rate in the opposite direction be *μf_e_* and the current GC-content be *f*. Then we expect the proportion of mutations that are S->W to be

$$x(f_{e},f)=\frac{f\mu (1-f_{e})}{f\mu (1-f_{e})+(1-f)\mu f_{e}}=\frac{f(1-f_{e})}{f(1-f_{e})+(1-f)f_{e}}$$ (1)

Let us assume that *f*_*e*_ is normally distributed. Then the likelihood of observing *i* S>W mutations out of a total of *n* S>W and w>S mutations is

$$L=\int_{0}^{1}N(f_{e};\bar{f_{e}},\sigma )B(n,i,x(f_{e},f))df_{e}/\int_{0}^{1}N(f_{e};\bar{f_{e}},\sigma )df_{e}$$ (2)

The total log-likelihood is therefore the sum of the log of Eq 2 for each MB or 100KB window across all the windows in the genome. The maximum likelihood values were obtained by manually searching for the ML values in *Mathematica*.

### Simulations

In a number of analyses, we simulate DNMs under assumed model; for example, using the 7-mer model of Aggarwala et al. [40]. In these simulations, we calculate the expected number of DNMs given the window’s mutation rate, the number of relevant sites and the total number of DNMs, and then generated a random Poisson variate from this expectation. In each simulation, we generated 1000 simulated datasets.

## Supporting information

S1 Table The CV of the gamma distribution fitted to the three datasets separately at the 100KB scale, along with the 96% confidence intervals. (DOCX)

S2 Table The CV of the gamma distribution fitted to the three datasets separately at the 100KB scale, along with the 96% confidence intervals. (DOCX)

S3 Table The correlation between different mutational types for the Francioli, Wong and Jonsson datasets at the 100KB scale. The expected correlations were estimated by simulation assuming a common distribution for two mutational categories. 100 simulations were conducted. * p<0.05, ** p<0.01, *** p<0.001.
(DOCX)

S4 Table Correlation between the density of DNMs and the mutation rate estimates from the mutation rate models of Aggarwala et al. and Michaelson et al. at the 100KB scale. *p < 0.05 **p < 0.01 *** p < 0.001.
(DOCX)

S5 Table Correlations between the density of DNMs and various genomic features at the 100KB scale. * p<0.05, ** p<0.01, ***p<0.001.
(DOCX)

S6 Table Correlations between the density of DNMs and the first two principle components of the genomic features at the 1MB scale. Note that DNM density is considered across the appropriate type of site (e.g. CpG C>T mutations at CpG sites). *p<0.05, **p<0.01, ***p<0.001.
(DOCX)

S7 Table The standardised regression coefficients from a stepwise multiple regression with forward variable selection (parameter has to be significant at p<0.05 to be added to the model) at the 100KB scale. (DOCX)

S8 Table Testing for the difference in slopes. Proportion of bootstrap replicates in which the slope of the normalised DNM density at 1MB scale versus recombination rate, is greater than the slope of the normalised number of substitutions (or SNPs) versus recombination rate. 100 bootstrap replicates were performed in each case. Results are shown for male and female specific recombination rates.
(DOCX)

S9 Table Testing for difference in slopes. Proportion of bootstrap replicates in which the slope of the normalised DNM density at 100KB versus sex-averaged recombination rate, is greater than the slope of the normalised number of substitutions (or SNPs) versus recombination rate. 100 bootstrap replicates were performed in each case.
(DOCX)

S10 Table The observed and expected correlations between DNM and SNP density at the 1MB scale for each category of mutation. The expected correlation is that expected if all the variation in SNP density is due to variation in the mutation rate; this was estimated by generating 100 simulated datasets. The p-values in the expected column are for the proportion of simulated datasets in which the correlation was significantly higher, or lower) than the observed correlation. *p<0.05, **p<0.01.
(DOCX)

S1 Fig The gamma distribution fitted to the three datasets at the 100KB scale. In order of decreasing variance: Blue: Francioli, Maroon: Wong: Olive: Jonsson.
(TIF)

S2 Fig Goodness of fit of the gamma distribution at 1MB scale. The distribution of observed and expected number of blocks with a given number of DNMs. The expected number was estimated using the fitted gamma distribution. A) Francioli, B) Wong, C) Jonsson.
(TIF)

S3 Fig Goodness of fit of the gamma distribution at 100KB scale. The distribution of observed and expected number of blocks with a given number of DNMs. The expected number was estimated using the fitted gamma distribution. A) Francioli, B) Wong, C) Jonsson.
(TIF)

S4 Fig The predicted equilibrium GC content versus the current GC content at the 100KB scale. Using mutation rates inferred from the Francioli (blue), Wong (orange) and Jonsson (green) DNMs. Several of the datapoints are coincident.
(TIF)

S5 Fig Divergence (number of substitutions per base pair) as a function of alignment length for two sets of alignments. A) UCSD pairwise alignments (PZ) and B) UCSD multiz alignments (MZ). Also given is the correlation coefficient and its significance.
(TIF)

S6 Fig Investigating why divergence is correlated to RR using male and female recombination rates at 1MB. The slope between normalised DNM density and normalised recombination rate (RR) (Wong—blue, Francioli–orange, Jonsson–grey), normalised substitution density and RR (yellow) and normalised SNP density and RR (light blue) at the 1MB scale. In each case the values were normalised by dividing the values by the mean. Panel A is for male recombination rates, panel B for female recombination rates.
(TIF)

S7 Fig Investigating why divergence is correlated to RR at 100KB. The slope between normalised DNM density and normalised recombination rate (RR) (Wong—blue, Francioli–orange, Jonsson—grey), normalised substitution density and RR (yellow) and normalised SNP density and RR (light blue) at the 100KB scale. In each case the values were normalised by dividing the values by the mean. Sex-averaged RRs were used.
(TIF)

S8 Fig The relationship between the log mutation rate (estimated as number of DNMs over number of sites) and the mutability index from Michaelson et al. (TIF)
