# Supplementary material for: Large scale variation in the rate of germ-line de novo mutation, base composition, divergence and diversity in humans
Source: PLoS Genet. 2018 Mar 28;14(3):e1007254. doi: 10.1371/journal.pgen.1007254 (PMC5891062; doi:10.1371/journal.pgen.1007254)
Supplement: S1 Table — (DOCX) [file pgen.1007254.s001.docx]

|  | Francioli 100KB | Wong 100KB | Jonsson 100KB |
| --- | --- | --- | --- |
| Ovarall | 0.33 (0.26, 0.40) | 0.34 (0.32, 0.38) | 0.21 (0.19, 0.22) |
| CpG | 0.54 (0.14, 0.79) | 0.43 (0.27, 0.57) | 0.29 (0.24, 0.33) |
| nonCpG | 0.35 (0.26, 0.43) | 0.33 (0.30, 0.37) | 0.22 (0.21, 0.24) |
| nonCpG ts | 0.33 (0.15, 0.45) | 0.29 (0.23, 0.35) | 0.19 (0.16, 0.22) |
| nonCpG tv | 0.55 (0.38, 0.69) | 0.40 (0.32, 0.48) | 0.32 (0.29, 0.35) |
| S>W | 0.38 (0.23, 0.49) | 0.38 (0.32, 0.43 | 0.23 (0.20, 0.25) |
| W>S | 0.077 (0, 0.087) | 0.29 (0.18, 0.37) | 0.16 (0.11, 0.20) |
