# Supplementary material for: Large scale variation in the rate of germ-line de novo mutation, base composition, divergence and diversity in humans
Source: PLoS Genet. 2018 Mar 28;14(3):e1007254. doi: 10.1371/journal.pgen.1007254 (PMC5891062; doi:10.1371/journal.pgen.1007254)
Supplement: S2 Table — (DOCX) [file pgen.1007254.s002.docx]

|  | 100KB | | | 1MB | | |
| --- | --- | --- | --- | --- | --- | --- |
| Test | Francioli | Wong | Jonsson | Francioli | Wong | Jonsson |
| CpG v nonCpG | ns | ns | 0.011 | <0.001 | ns | <0.001 |
| nonCpG ts v tv | 0.031 | 0.025 | <0.001 | ns | ns | <0.001 |
| S>W v W>S | ns | ns | 0.0081 | 0.019 | ns | 0.0062 |
