# Supplementary material for: Large scale variation in the rate of germ-line de novo mutation, base composition, divergence and diversity in humans
Source: PLoS Genet. 2018 Mar 28;14(3):e1007254. doi: 10.1371/journal.pgen.1007254 (PMC5891062; doi:10.1371/journal.pgen.1007254)
Supplement: S3 Table — The expected correlations were estimated by simulation assuming a common distribution for two mutational categories. 100 simulations were conducted. * p<0.05, ** p<0.01, *** p<0.001. (DOCX) [file pgen.1007254.s003.docx]

|  | Observed | Expected | Expected < observed |
| --- | --- | --- | --- |
| *Francioli* |  |  |  |
| CpG v nonCpG | 0.018*** | 0.024 | 0.18 |
| Non CpG ts v tv | 0.015* | 0.024 | 0.07 |
| S>W v W>S | 0.012* | 0.0014 | 0.98 |
|  |  |  |  |
| *Wong* |  |  |  |
| CpG v nonCpG | 0.029*** | 0.041 | 0.06 |
| Non CpG ts v tv | 0.050*** | 0.042 | 0.87 |
| S>W v W>S | 0.032*** | 0.041 | 0.09 |
|  |  |  |  |
| *Jonsson* |  |  |  |
| CpG v nonCpG | 0.044*** | 0.073 | 0.0 |
| Non CpG ts v tv | 0.076*** | 0.069 | 0.92 |
| S>W v W>S | 0.039*** | 0.049 | 0.02 |
