# Supplementary material for: Large scale variation in the rate of germ-line de novo mutation, base composition, divergence and diversity in humans
Source: PLoS Genet. 2018 Mar 28;14(3):e1007254. doi: 10.1371/journal.pgen.1007254 (PMC5891062; doi:10.1371/journal.pgen.1007254)
Supplement: S4 Table — *p < 0.05 **p < 0.01 *** p < 0.001. (DOCX) [file pgen.1007254.s004.docx]

|  | Aggarwala | | Michaelson | |
| --- | --- | --- | --- | --- |
|  | Observed | Simulated | Observed | Simulated |
| Francioli | -0.057*** | 0.060*** | 0.031*** | 0.19*** |
| Wong | 0.090*** | 0.094 | 0.049*** | 0.29*** |
| Jonsson | 0.043*** | 0.18*** | 0.044*** | 0.49*** |
