# Supplementary material for: Large scale variation in the rate of germ-line de novo mutation, base composition, divergence and diversity in humans
Source: PLoS Genet. 2018 Mar 28;14(3):e1007254. doi: 10.1371/journal.pgen.1007254 (PMC5891062; doi:10.1371/journal.pgen.1007254)
Supplement: S5 Table — * p<0.05, ** p<0.01, ***p<0.001. (DOCX) [file pgen.1007254.s005.docx]

|  | Francioli | Wong | Jonsson | W<>W & S<>S subs |
| --- | --- | --- | --- | --- |
| Male recombination rate | 0.023** | 0.079*** | 0.038*** | 0.125*** |
| Female recombination rate | 0.017* | 0.084*** | 0.030*** | 0.082*** |
| H3K4me1 | -0.017* | 0.082*** | 0.005 | -0.224*** |
| H3K4me3 | -0.041*** | -0.003 | -0.023** | -0.243*** |
| H3K27me3 | -0.015* | 0.023** | -0.004 | -0.083*** |
| H3K27ac | -0.024*** | 0.048*** | -0.004 | -0.246*** |
| Transcription rate | -0.031*** | 0.004 | 0.008 | -0.084*** |
| H3K4me1PB | -0.030*** | 0.035*** | -0.024*** | -0.226*** |
| H3K9me3PB | 0.039*** | -0.055*** | 0.017* | 0.212*** |
| Nucleosome occupancy | -0.005 | 0.110*** | 0.020** | -0.210*** |
| DNAse hypersensitivity | -0.039*** | 0.06*** | 0.008 | -0.206*** |
| Replication time | -0.057*** | 0.011 | -0.033*** | -0.362*** |
| GC content | -0.031*** | 0.094*** | 0.022** | -0.217*** |
