# Supplementary material for: Large scale variation in the rate of germ-line de novo mutation, base composition, divergence and diversity in humans
Source: PLoS Genet. 2018 Mar 28;14(3):e1007254. doi: 10.1371/journal.pgen.1007254 (PMC5891062; doi:10.1371/journal.pgen.1007254)
Supplement: S6 Table — Note that DNM density is considered across the appropriate type of site (e.g. CpG C>T mutations at CpG sites). *p<0.05, **p<0.01, ***p<0.001. (DOCX) [file pgen.1007254.s006.docx]

|  | Francioli | | Wong | | Jonsson | |
| --- | --- | --- | --- | --- | --- | --- |
|  | PC1 | PC2 | PC1 | PC2 | PC1 | PC2 |
| CpG C>T | -0.17*** | 0.062** | -0.049* | 0.13*** | -0.25*** | 0.099*** |
| CpG C>A | -0.030 | 0.044* | 0.009 | 0.019 | -0.044* | 0.028 |
| CpG C>G | -0.015 | 0.029 | 0.015 | 0.0040 | -0.050* | 0.014 |
| non C>T | -0.081*** | 0.092*** | 0.043* | 0.14*** | -0.121*** | 0.048* |
| non C>A | -0.10*** | -0.003 | -0.078*** | 0.092*** | -0.224*** | 0.054* |
| non C>G | -0.057** | 0.047* | -0.010 | 0.094*** | -0.090*** | 0.025 |
| non T>C | -0.11*** | 0.019 | 0.042* | 0.13*** | -0.121*** | 0.027 |
| non T>G | -0.029 | -0.019 | 0.033 | 0.057** | -0.118*** | 0.011 |
| non T>A | -0.078*** | 0.051* | -0.060** | 0.035 | -0.148*** | 0.031 |
