# Supplementary material for: Large scale variation in the rate of germ-line de novo mutation, base composition, divergence and diversity in humans
Source: PLoS Genet. 2018 Mar 28;14(3):e1007254. doi: 10.1371/journal.pgen.1007254 (PMC5891062; doi:10.1371/journal.pgen.1007254)
Supplement: S7 Table — (DOCX) [file pgen.1007254.s007.docx]

|  | Francioli | Wong | Jonsson | W<>W & S<>S subs |
| --- | --- | --- | --- | --- |
| Male recombination rate | 0.019*** | 0.047*** | 0.028*** | 0.095*** |
| Female recombination rate |  |  |  | 0.068*** |
| H3K4me1 | 0.027** | 0.049*** |  | 0.036*** |
| H3K4me3 |  | -0.049*** | -0.025** |  |
| H3K27me3 |  |  |  | -0.075*** |
| H3K27ac |  | 0.033* |  |  |
| Transcription rate | -0.014* |  |  |  |
| H3K4me1PB |  |  | -0.024** | -0.069*** |
| H3K9me3PB | -0.002 | -0.078*** | 0.073*** | 0.28*** |
| Nucleosome occupancy | 0.055*** | 0.16*** | -0.082*** | -0.50*** |
| DNAse hypersensitivity |  | 0.057*** | -0.046** | -0.26*** |
| Rep time | -0.060*** | -0.072*** | -0.033*** | -0.25*** |
| GC content | -0.062** | -0.12*** | 0.20*** | 0.70*** |
| r^2^ | 0.006 | 0.022 | 0.007 | 0.20 |
