# Supplementary material for: Large scale variation in the rate of germ-line de novo mutation, base composition, divergence and diversity in humans
Source: PLoS Genet. 2018 Mar 28;14(3):e1007254. doi: 10.1371/journal.pgen.1007254 (PMC5891062; doi:10.1371/journal.pgen.1007254)
Supplement: S8 Table — Proportion of bootstrap replicates in which the slope of the normalised DNM density at 1MB scale versus recombination rate, is greater than the slope of the normalised number of substitutions (or SNPs) versus recombination rate. 100 bootstrap replicates were performed in each case. Results are shown for male and female specific recombination rates. (DOCX) [file pgen.1007254.s008.docx]

| DNM data | Subs or SNPs | SW | WS | SSWW |
| --- | --- | --- | --- | --- |
| *Male RR* |  |  |  |  |
| Francioli | Subs | 0.96 | 0 | 0.93 |
| Wong | Subs | 1 | 0.09 | 0.78 |
| Jonsson | Subs | 1 | 0 | 0.01 |
| Francioli | SNPs | 0.61 | 0 | 0.97 |
| Wong | SNPs | 1 | 1 | 0.90 |
| Jonsson | SNPs | 0.2 | 0 | 0 |
|  |  |  |  |  |
| *Female RR* |  |  |  |  |
| Francioli | Subs | 1 | 0 | 0.57 |
| Wong | Subs | 1 | 0.23 | 0.9 |
| Jonsson | Subs | 1 | 0 | 0 |
| Francioli | SNPs | 0.75 | 0 | 0.65 |
| Wong | SNPs | 1 | 1 | 0.96 |
| Jonsson | SNPs | 0 | 0 | 0.01 |
