# Supplementary material for: Large scale variation in the rate of germ-line de novo mutation, base composition, divergence and diversity in humans
Source: PLoS Genet. 2018 Mar 28;14(3):e1007254. doi: 10.1371/journal.pgen.1007254 (PMC5891062; doi:10.1371/journal.pgen.1007254)
Supplement: S9 Table — Proportion of bootstrap replicates in which the slope of the normalised DNM density at 100KB versus sex-averaged recombination rate, is greater than the slope of the normalised number of substitutions (or SNPs) versus recombination rate. 100 bootstrap replicates were performed in each case. (DOCX) [file pgen.1007254.s009.docx]

|  | SW | WS | SSWW |
| --- | --- | --- | --- |
| *Substitutions* |  |  |  |
| Francioli | 1 | 0 | 0.54 |
| Wong | 1 | 0.1 | 1 |
| Jonsson | 1 | 0 | 0 |
|  |  |  |  |
| *SNPs* |  |  |  |
| Francioli | 0.85 | 0.02 | 0.58 |
| Wong | 1 | 1 | 1 |
| Jonsson | 0.09 | 0 | 0 |
