# Supplementary material for: Large scale variation in the rate of germ-line de novo mutation, base composition, divergence and diversity in humans
Source: PLoS Genet. 2018 Mar 28;14(3):e1007254. doi: 10.1371/journal.pgen.1007254 (PMC5891062; doi:10.1371/journal.pgen.1007254)
Supplement: S10 Table — The expected correlation is that expected if all the variation in SNP density is due to variation in the mutation rate; this was estimated by generating 100 simulated datasets. The p-values in the expected column are for the proportion of simulated datasets in which the correlation was significantly higher, or lower) than the observed correlation. *p<0.05, **p<0.01. (DOCX) [file pgen.1007254.s010.docx]

|  | Francioli | | Wong | | Jonsson | |
| --- | --- | --- | --- | --- | --- | --- |
|  | Obs. | Exp. | Obs. | Exp. | Obs. | Exp. |
| 1MB |  |  |  |  |  |  |
| All | 0.18** | 0.24** | 0.31** | 0.35 | 0.43** | 0.58** |
| CpG C>T | 0.20** | 0.11** | 0.10** | 0.14** | 0.32** | 0.30 |
| non C>T | 0.13** | 0.11 | 0.12** | 0.17* | 0.23** | 0.31** |
| non C>A | 0.12** | 0.12 | 0.15** | 0.19* | 0.30** | 0.31 |
| non C>G | 0.10** | 0.16* | 0.31** | 0.25** | 0.35** | 0.44** |
| non T>C | 0.10** | 0.11 | 0.11** | 0.16* | 0.20** | 0.29** |
| non T>G | 0.0036 | 0.072** | 0.073** | 0.11 | 0.18** | 0.21 |
| non T>A | 0.090** | 0.084 | 0.081** | 0.13* | 0.20** | 0.25* |
|  |  |  |  |  |  |  |
| 100KB |  |  |  |  |  |  |
| All | 0.064** | 0.083** | 0.12** | 0.13 | 0.17** | 0.24** |
| CpG C>T | 0.068** | 0.046** | 0.043** | 0.064** | 0.14** | 0.14 |
| non C>T | 0.042** | 0.044 | 0.047** | 0.069** | 0.089** | 0.13** |
| non C>A | 0.047** | 0.045 | 0.044** | 0.074** | 0.11** | 0.12 |
| non C>G | 0.031** | 0.056* | 0.11** | 0.089* | 0.12** | 0.17** |
| non T>C | 0.041** | 0.039 | 0.034** | 0.063** | 0.075** | 0.12** |
| non T>G | 0.0064** | 0.029** | 0.023** | 0.043* | 0.052** | 0.082** |
| non T>A | 0.025** | 0.032 | 0.019** | 0.048** | 0.066** | 0.095** |
