# Supplementary figures and images for: Large scale variation in the rate of germ-line de novo mutation, base composition, divergence and diversity in humans
Source: PLoS Genet. 2018 Mar 28;14(3):e1007254. doi: 10.1371/journal.pgen.1007254 (PMC5891062; doi:10.1371/journal.pgen.1007254)

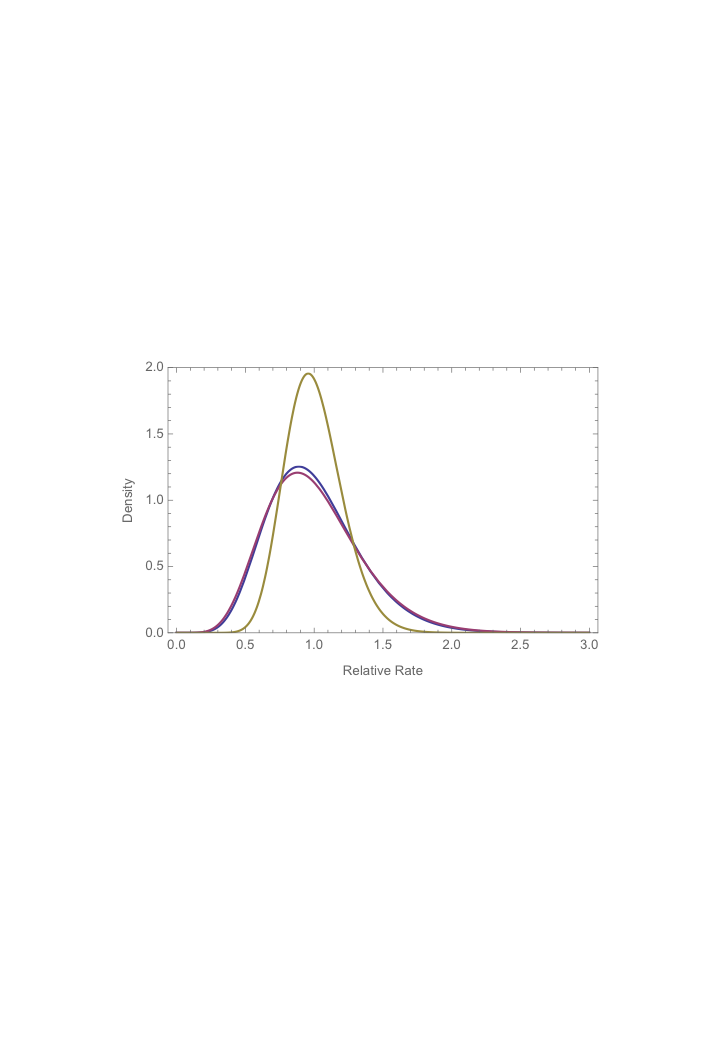

Supplement: S1 Fig — In order of decreasing variance: Blue: Francioli, Maroon: Wong: Olive: Jonsson. (TIF) [file pgen.1007254.s011.tif]

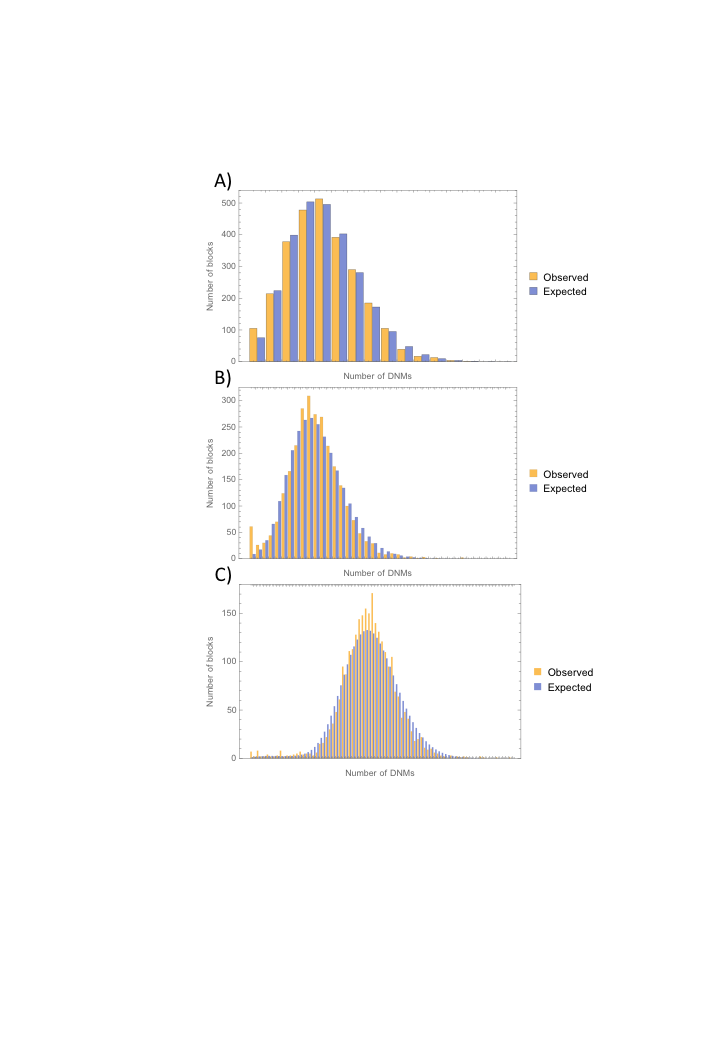

Supplement: S2 Fig — The distribution of observed and expected number of blocks with a given number of DNMs. The expected number was estimated using the fitted gamma distribution. A) Francioli, B) Wong, C) Jonsson. (TIF) [file pgen.1007254.s012.tif]

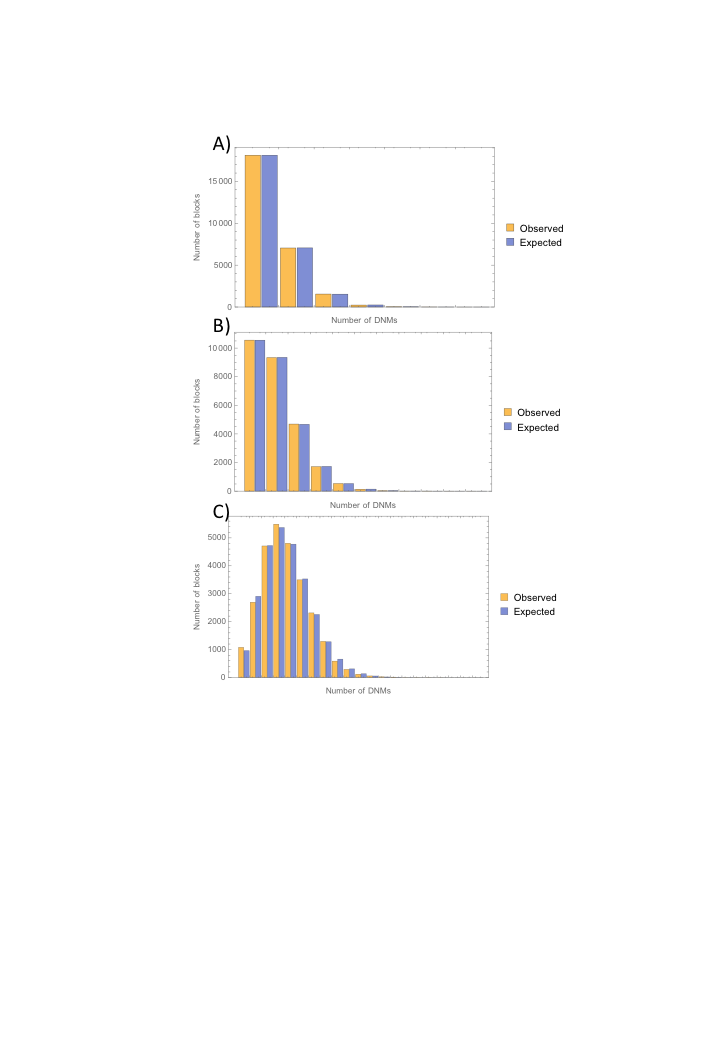

Supplement: S3 Fig — The distribution of observed and expected number of blocks with a given number of DNMs. The expected number was estimated using the fitted gamma distribution. A) Francioli, B) Wong, C) Jonsson. (TIF) [file pgen.1007254.s013.tif]

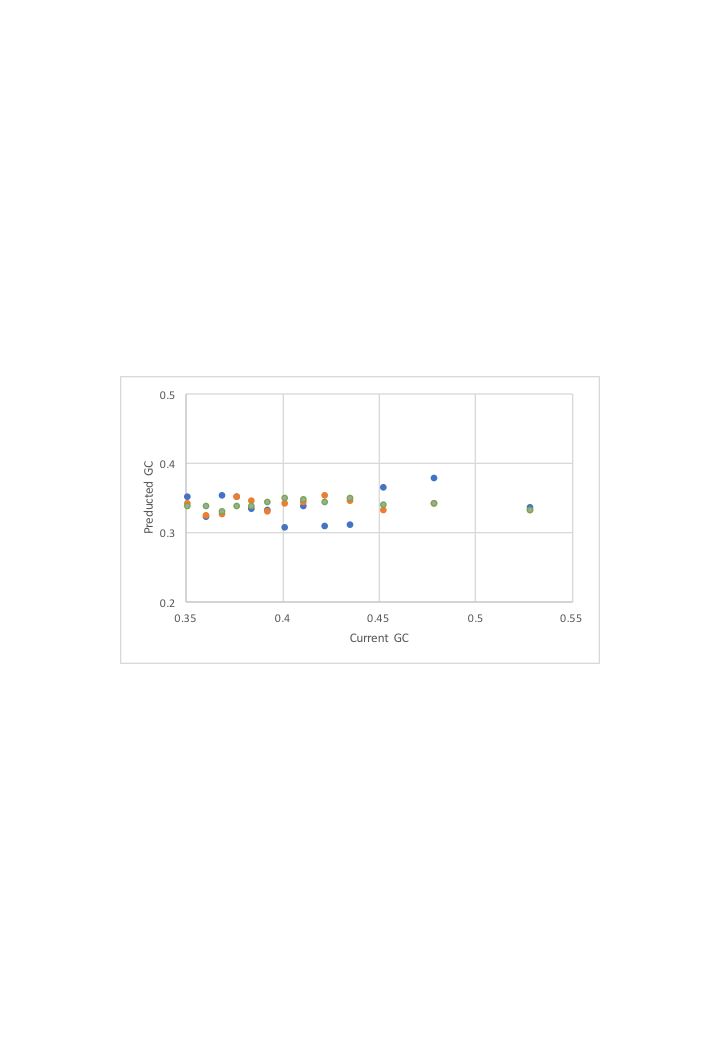

Supplement: S4 Fig — Using mutation rates inferred from the Francioli (blue), Wong (orange) and Jonsson (green) DNMs. Several of the datapoints are coincident. (TIF) [file pgen.1007254.s014.tif]

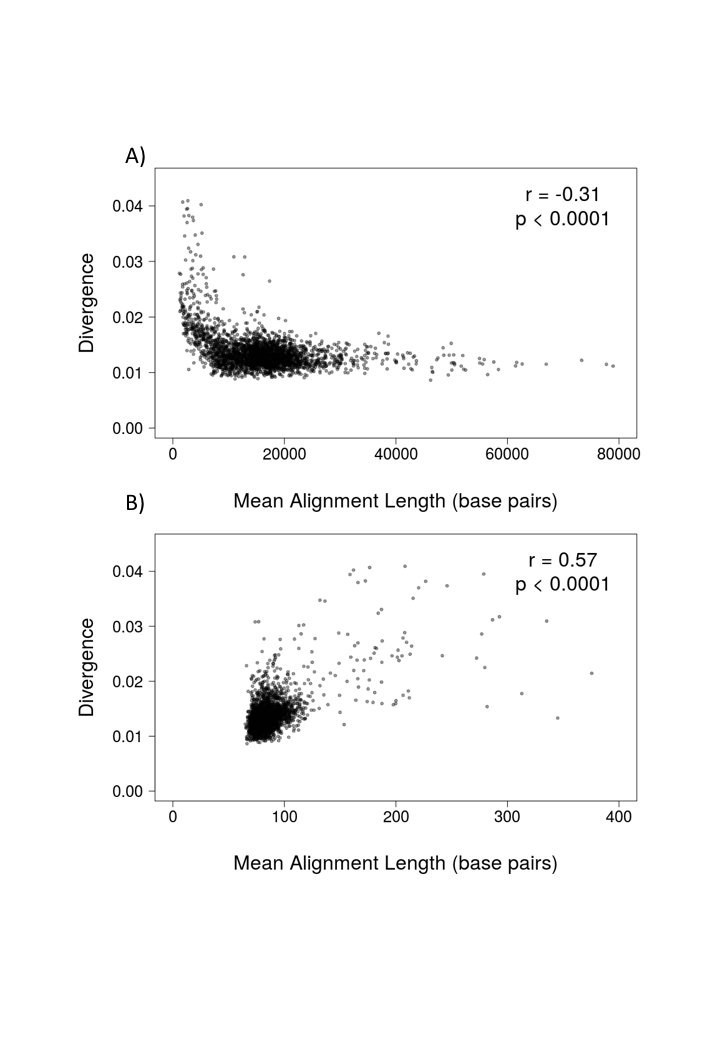

Supplement: S5 Fig — A) UCSD pairwise alignments (PZ) and B) UCSD multiz alignments (MZ). Also given is the correlation coefficient and its significance. (TIF) [file pgen.1007254.s015.tif]

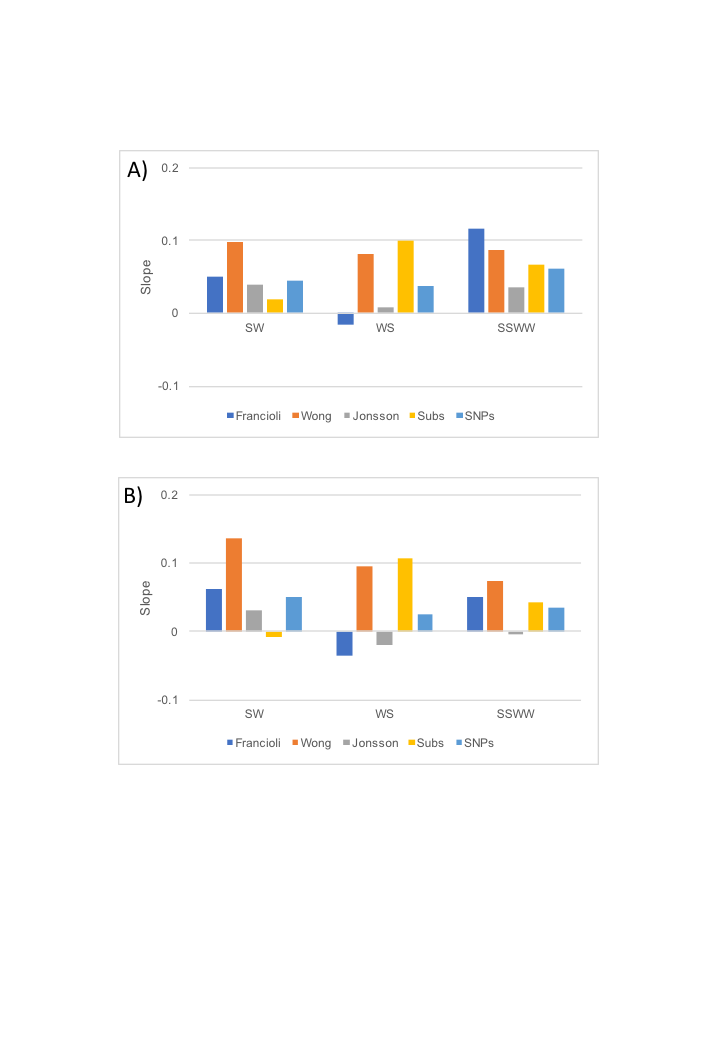

Supplement: S6 Fig — The slope between normalised DNM density and normalised recombination rate (RR) (Wong—blue, Francioli–orange, Jonsson–grey), normalised substitution density and RR (yellow) and normalised SNP density and RR (light blue) at the 1MB scale. In each case the values were normalised by dividing the values by the mean. Panel A is for male recombination rates, panel B for female recombination rates. (TIF) [file pgen.1007254.s016.tif]

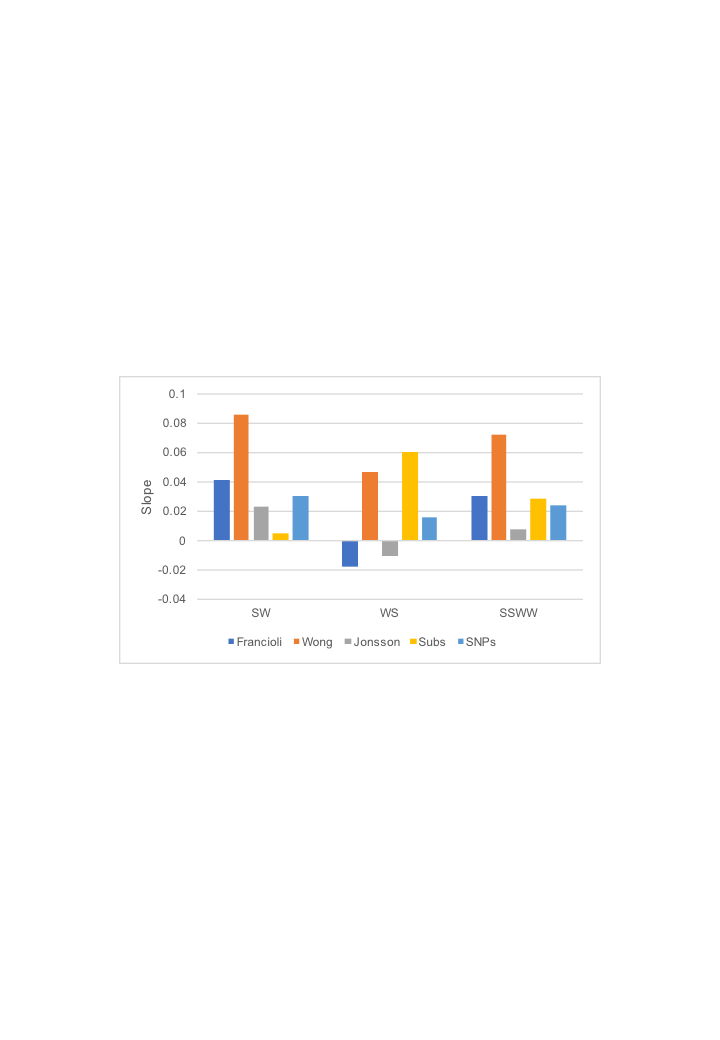

Supplement: S7 Fig — The slope between normalised DNM density and normalised recombination rate (RR) (Wong—blue, Francioli–orange, Jonsson—grey), normalised substitution density and RR (yellow) and normalised SNP density and RR (light blue) at the 100KB scale. In each case the values were normalised by dividing the values by the mean. Sex-averaged RRs were used. (TIF) [file pgen.1007254.s017.tif]

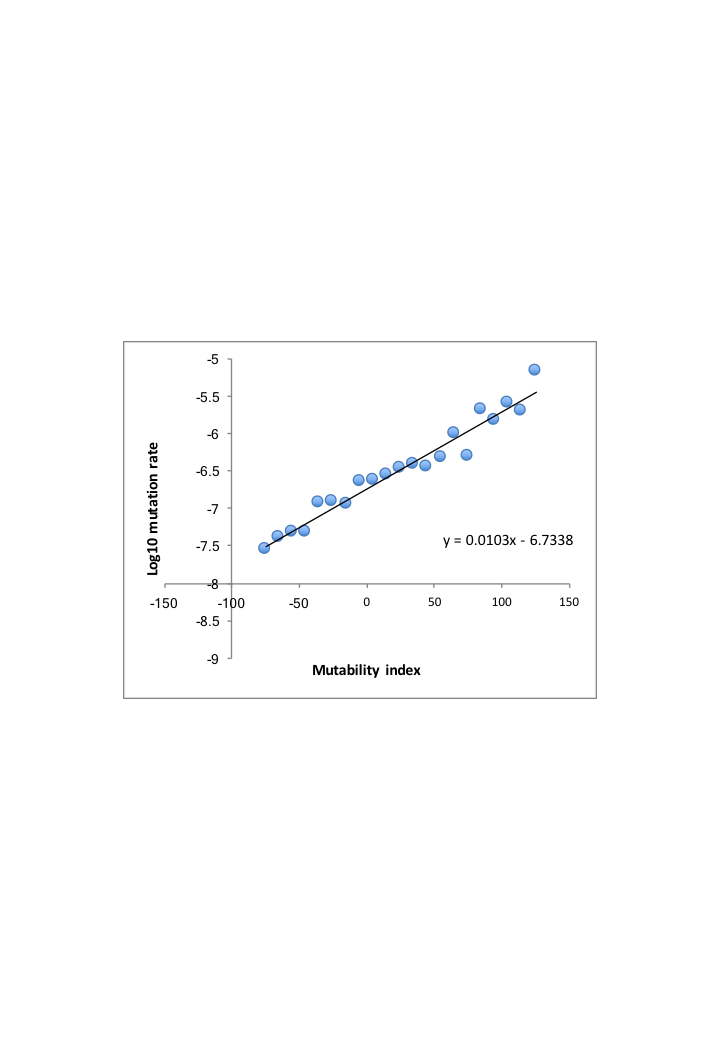

Supplement: S8 Fig — (TIF) [file pgen.1007254.s018.tif]
